# Guinea baboons are strategic cooperators

**DOI:** 10.1126/sciadv.adi5282

**Published:** 2023-10-27

**Authors:** Anthony Formaux, Dan Sperber, Joël Fagot, Nicolas Claidière

**Affiliations:** ^1^ Laboratoire de Psychologie Cognitive, UMR7290, Université Aix-Marseille/CNRS, Marseille, France.; ^2^ Station de Primatologie-Celphedia, CNRS UAR846, Rousset, France.; ^3^ Central European University, Wien, Austria.; ^4^ Institut Jean Nicod, Ecole Normale Supérieure, Paris, France.; ^5^ Institute for Language, Communication and the Brain, Université Aix-Marseille, CNRS, Aix-en-Provence, France.

## Abstract

Humans are strategic cooperators; we make decisions on the basis of costs and benefits to maintain high levels of cooperation, and this is thought to have played a key role in human evolution. In comparison, monkeys and apes might lack the cognitive capacities necessary to develop flexible forms of cooperation. We show that Guinea baboons (*Papio papio*) can use direct reciprocity and partner choice to develop and maintain high levels of cooperation in a prosocial choice task. Our findings demonstrate that monkeys have the cognitive capacities to adjust their level of cooperation strategically using a combination of partner choice and partner control strategies. Such capacities were likely present in our common ancestor and would have provided the foundations for the evolution of typically human forms of cooperation.

## Teaser

Guinea baboons can fine tune their behavior according to the cost and benefits of their action to maintain cooperation.

## INTRODUCTION

Humans can readily solve new cooperative problems by fine-tuning their level of cooperation depending on the costs and benefits of their actions (*1*–*3*) [following others, we define cooperation as any behavior or trait that procures a benefit to another individual (*4*, *5*)]. When confronted with a noncooperative partner, humans will typically try to find another more cooperative one [partner choice strategies (*6*–*8*)] and/or try to increase the cooperativeness of the partner (partner control strategies) using, for instance, punishment (*9*) or a “tit-for-tat” strategy (*10*, *11*), also known as direct reciprocity.

The evolutionary origin of these aptitudes for strategic cooperation is still unclear, and the extent to which they are shared with other primates is debated (*12*). While it is now clear that nonhuman primates (NHPs) can exhibit many forms of cooperation in the wild (*13*–*16*), the lack of a controlled environment that can provide clear information about the origin of cooperation and the reward structure of different actions limit the understanding of strategies. To address this challenge, experimental paradigms have been developed to better understand how stable cooperation could emerge in novel situations. However, 20 years of research have shown that NHP experience difficulties in solving simple cooperative tasks (*17*), raising the possibility that they could lack the cognitive mechanisms necessary to develop flexible and adaptive cooperation (*18*–*21*).

**Fig. 1.**
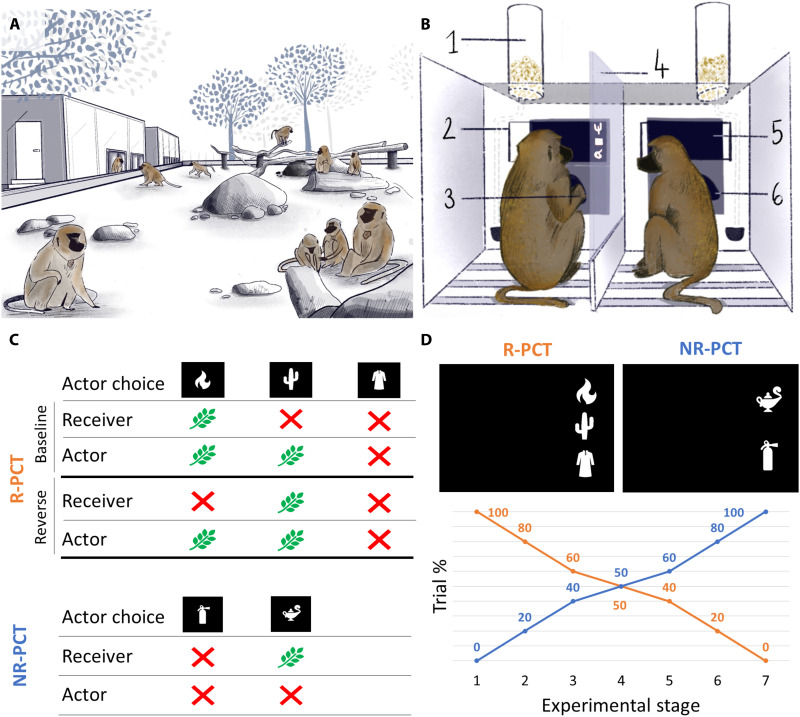
Experimental material and design. (**A**) Eighteen Guinea baboons living in an enclosure have ad libitum access to trailers containing five S-ALDM (social automated learning device for monkeys). (**B**) Detail of the organization of one S-ALDM, where two monkeys can participate in experiments side by side while seeing each other’s behavior. 1, reward dispenser; 2, viewing window; 3, RFID microchip; 4, transparent partition; 5, touchscreen. 6, reaching hole with RFID reader for automatic identification. (**C**) Illustration of the possible outcomes of a trial performed by the actor during experiments 1 and 2. For rewarded prosocial choice trials (R-PCT), the stimuli used were the same for both baseline and reversal phase, but the outcomes were different. During NR-PCT trials, introduced during experiment 2, the actor was never rewarded. (**D**) During experiment 2, NR-PCT trials were progressively introduced among R-PCT ones. Each experimental stage lasted 2 days. Illustrations by L. Rivoal.

Experiments that are performed in a group context, in which an entire group of individuals can freely participate in cooperative tasks, have the advantage of facilitating the emergence of cooperative strategies, and some show evidence of cooperation (*22*), although not always (*23*). However, because individuals are free to interact and decisions potentially involve many factors (such as relatedness, prior interactions, short- and long-term relationships, etc.), the strategies are difficult to disentangle. On the other hand, experiments with isolated pairs of individuals could provide clearer evidence, but they usually do not give rise to high levels of cooperation (*17*, *24*–*26*), maybe because they limit the opportunities for strategic cooperation by limiting the choices of individuals (*27*). Here, we performed experiments in a group context in which individuals could freely choose their partner (Fig. 1, A and B), but individuals took part in a cooperation task in pairs, through an automatized touchscreen system that recorded the full history of interactions between individuals and provided a clearly specified reward structure (*28*–*31*). The results of the three experiments that we present below correspond to 95 days of testing in a group of 18 Guinea baboons and a total of 248,616 trials in which 153 different pairs of individuals interacted, providing a unique window into the emergence and stabilization of cooperation and showing that some Guinea baboons were able to use both partner choice and reciprocity to stabilize high levels of cooperation.

## RESULTS

### Guinea baboons develop and sustain prosociality

**Fig. 2.**
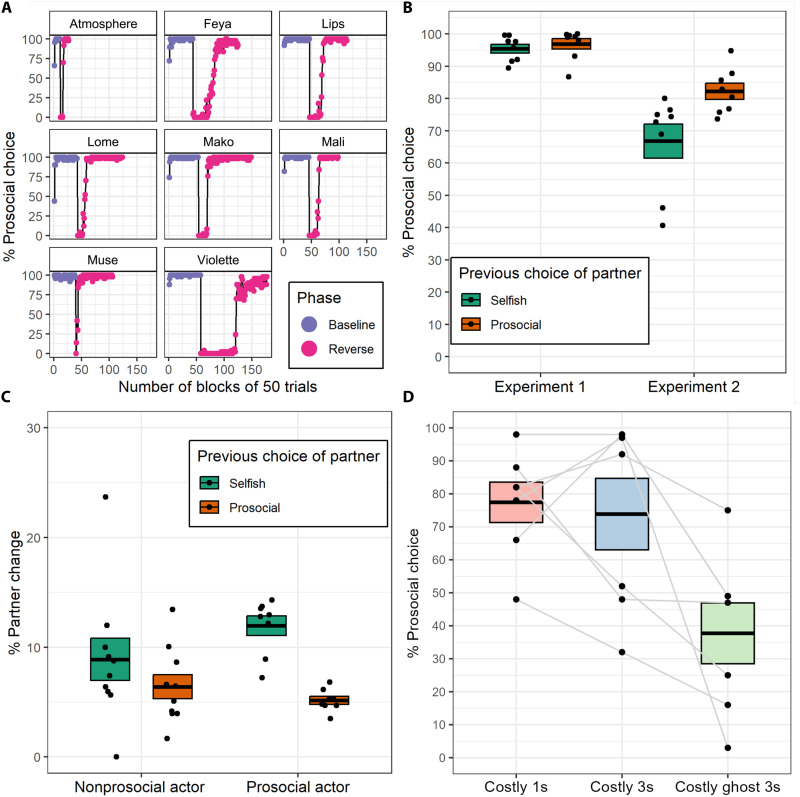
Prosocial baboons’ choices and strategies along the experiments. (**A**) Proportion of prosocial choices in each block of 50 trials during the baseline phase (purple) and the reversal phase (pink) of experiment 1 for the eight prosocial individuals. (**B**) Average prosocial choice of the eight prosocial individuals in experiments 1 (R-PCT trials) and 2 (NR-PCT trials only) depending on the choice of their partner in the previous trial. We consider the succession of two trials, less than 15 s apart, in which one prosocial individual (P) is paired with another individual (I). On the first trial, I is the actor, P is the receiver, and vice versa for the second trial. (**C**) Proportion of trials with a partner change in experiment 2 depending on the partner choice during the previous trial. We considered a change of partner when a prosocial individual performed a trial with a different partner than the previous trial. (**D**) Proportion of prosocial choice by prosocial monkeys during experiment 3. During experiment 3, the prosocial stimuli needed to be touched twice, first with a 1-s delay, then with a 3-s delay, and lastly with a 3-s delay in a ghost condition with no partner present. (B to D) Box plots represent means ± SE.

During experiment 1, eight individuals reached our predefined criteria (*28*, *32*) of at least 80% prosocial choice in one block of 50 trials in both the baseline and reversed condition when a partner was present. These eight individuals therefore showed a flexible adaptation to the change in the stimuli outcome, changing their choice of stimuli when the contingencies of the task were reversed. By contrast, during the ghost control, none of the 18 baboons changed their behavior during the reversed phase (a significant difference from the test condition: binomial test, 0 of 18 versus 8 of 18, *P* < 0.001; additional analysis is in Supplementary Text; all reported tests are two-sided). This shows that Guinea baboons adapted their choice of stimuli in the presence of a partner but not in the absence of one. During the test condition, in their last block of 50 reversed trials, the eight prosocial monkeys reached an average proportion of prosocial choice of 98% (SD = 2.6, min = 92, max = 100; Fig. 2A). Our results therefore show that monkeys can develop and sustain high levels of cooperation in the prosocial choice task (see figs. S3 and S4 for more details about the other baboons).

### Without direct benefit, Guinea baboons remain prosocial

During experiment 2, we wanted to challenge the baboons’ capacity to maintain cooperation by gradually introducing nonrewarded prosocial choice task (NR-PCT) trials. In these trials, the actor was never rewarded, one stimulus rewarded only the receiver (0-1), and the other did not (0-0) (Fig. 1, C and D). We progressively increased the proportion of NR-PCT trials with the rewarded PCT (R-PCT) trials to give monkeys time to adapt to the new nonreinforcement contingencies for the actor. Crucially, at the end of experiment 2, only NR-PCT trials remained and actors could therefore no longer receive rewards directly. The results showed that for the NR-PCT trials, the eight prosocial monkeys of experiment 1 all reached our criteria of 80% prosocial choice on 50 trials. In their last block of NR-PCT trials, these eight individuals reached a median proportion of prosocial choice of 83% (SD = 5.1, min = 78, max = 92, N trial = 50; see fig. S5).

Remember that, when only NR-PCT trials remained, the actor could either pair with a prosocial partner or avoid partners and wait for a solo task. Therefore, two prosocial monkeys paired together could only have a 50% reinforcement rate, on average, in experiment 2, when this would have been 100% in experiment 1. In humans, this situation would give rise to partner choice (*6*, *7*) and/or partner control through direct reciprocity (*10*, *11*) or punishment (*9*).

### Guinea baboons use reciprocity and partner choice

Regarding partner control strategies, the very high rate of prosocial choice (almost 100%) in experiment 1 suggested that the monkeys were choosing the prosocial option whether their partners also chose the prosocial option or not (Fig. 2B and fig. S6). As expected, the eight prosocial individuals made their choice regardless of their partner’s previous behavior after they had reached 80% prosocial choice [Generalized Linear Mixed Model (GLMM): β = 0.28, SE = 0.15, *z* = 1.83, *P* = 0.07]. However, in experiment 2, the same individuals were more likely to make the prosocial choice during NR-PCT trials if their partner had previously made a prosocial choice in either an R-PCT or NR-PCT trial (GLMM: β = 0.91, SE = 0.16, *z* = 5.81, *P* < 0.001; Fig. 2B and fig. S7), a form of direct reciprocity or tit-for-tat strategy.

In addition, we found that in both experiments, prosocial monkeys were more likely than nonprosocial monkeys to change partner when their partner chose the selfish stimuli in the previous trial (experiment 1: β_interaction_ = −0.34, SE = 0.07, *z* = −5.07, *P* < 0.001; experiment 2: β_interaction_ = −0.54, SE = 0.12, *z* = −4.59, *P* < 0.001). This form of partner choice existed in both prosocial and nonprosocial monkeys but was significantly stronger in the former (Fig. 2C and fig. S8). This partner choice created a situation in which there was a positive correlation between the number of trials performed by a pair of individuals and their joint level of prosociality (experiment 1: Spearman ρ = 0.31, *P* < 0.001; experiment 2: Spearman ρ = 0.32, *P* < 0.001; fig. S9).

Last, we also found that for prosocial monkeys, the rate of interrupted trial almost doubled between the two experiments (see Supplementary Text for details; seven percent of interrupted trial in experiment 1 and 13% in experiment 2). Interrupted trials could occur at two moments during the experiment, either during the fixation cross, marking the start of a trial, or during the choice screen, when the actor had to choose between the stimuli (fig. S10). Prosocial and nonprosocial monkeys kept a low rate of interruption during experiment 1, identical between the fixation cross and the choice screen, and when the monkey was an actor or a receiver, indicating a baseline level of interruption (β_intercept_ = −0.22, SE = 0.23). However, during experiment 2, while other interruption rates stayed constant, the actors’ rate of interruption during the choice screen greatly increased for prosocial monkeys (β_interaction_ = 0.54, SE = 0.14, *z* = 3.83, *P* < 0.001; fig. S11). Because monkeys tended to change partners more often after an interrupted trial (experiment 1: β = 1.32 SE = 0.03, *z* = 52.5, *P* < 0.001; experiment 2: β = 1.15, SE = 0.03, *z* = 33.26, *P* < 0.001; fig. S12) and were more likely to interrupt a trial when the partner had previously chosen a selfish response (experiment 1: β = 0.26 SE = 0.03, *z* = 9.96, *P* < 0.001; experiment 2: β = 0.17, SE = 0.05, *z* = 3.10, *P* = 0.002, fig. S13), this shows another strategy: When their partner did not make the prosocial choice, prosocial monkeys refused to make a decision when their turn came (i.e. interrupted the trial) and went looking for another partner.

Together, our results show that monkeys can be strategic cooperators using partner choice and partner control to develop and stabilize cooperation. During the first, less demanding experiment, they only used partner choice: changing partners more frequently when the partner did not make the prosocial choice. In the more demanding experiment 2, prosocial monkeys developed two additional response strategies when paired with a previously nonprosocial partner: They more frequently chose the selfish stimulus, and they were more likely to not respond at all, interrupting the trial and leading to a partner change.

### Guinea baboons can pay a cost to maintain cooperation

Experiment 2 suggested that baboons could pay a cost to maintain cooperation because they continued to select prosocial stimuli in the absence of reward to themselves. Experiment 3 further explored this possibility by including a direct cost to the prosocial choice. In a preliminary nonsocial task, we first explored whether monkeys could distinguish between a more costly stimulus that needed to be touched twice with a variable delay between the two touches and a less costly stimulus that needed to be touched only once (fig. S14). Our results showed that a delay of 1 s already represented a substantial cost that they would avoid (see Supplementary Text for details). We then performed a PCT similar to experiment 1 with the exception that the prosocial stimuli now needed to be touched twice, with a variable delay between the two touches (fig. S15). We performed a condition with a 1-s delay, followed by a 3-s delay, followed by a ghost control condition with no partner and a 3-s delay. We found that all seven prosocial monkeys (one prosocial monkey did not complete the experiment) were much more likely to choose the prosocial option in the 1- and 3-s delay condition, compared to the 3-s delay ghost control condition (β_1sec_ = 2.34, SE = 0.05, *z* = 50.4, *P* < 0.001; β_3sec_ = 2.11, SE = 0.05, *z* = 46.6, *P* < 0.001; fig. S16). This result demonstrates that monkeys are willing to pay a cost (pressing twice with a short delay) to sustain cooperation, a cost they are not ready to pay when there is no partner present [see (*33*) for a related finding with chimpanzees].

## DISCUSSION

The cognitive mechanisms underlying the development and maintenance of cooperative behavior in NHP primates remain unclear, and 20 years of experiments have yielded mixed results regarding the capacity of NHPs to cooperate in the prosocial choice task (*24*–*26*, *34*). By contrast, our results show that Guinea baboons can learn to cooperate and develop stable and recurrent prosocial behaviors to maximize their gains in a free-association turn-based environment. Eight individuals initially reached levels of prosocial choice rarely achieved in NHPs but frequent among humans (*35*, *36*). Ten individuals did not show evidence of prosocial behavior. We suspect that this is at least partly the result of a lack of understanding of a link between an action on one touchscreen system and its consequences on the other. The connection can be difficult to make because most of the time the baboons are performing individual tasks in which their actions have no consequence on the other systems. Supporting this hypothesis, we found that the same 10 individuals were not successful in a previous experiment in which they had to respond on the basis of information displayed on their partner’s screen (*37*), suggesting that they did not make the connection between the two systems in the current research.

Our results also demonstrate that Guinea baboons can adapt their strategy on the basis of costs and benefits and use partner control (direct reciprocity) and partner choice to maintain cooperation. By contrast, we found no systematic effect of the relationship between the actor and the partner on the behavior of prosocial individuals; in particular, dominance and social affiliation had no discernible effects (see Supplementary Text and figs. S17 and S18). This result is in line with a previous study showing that chimpanzees were sensitive to direct reciprocity in a prosocial choice task (*38*) but contrasts with some previous studies reporting a small effect of dominance and social affiliation in a similar task but lacking opportunities for reciprocity (*39*, *40*). Note that baboons first used partner choice and developed partner control only when the task became more demanding. This result is in accordance with recent suggestions that partner choice is the most common mechanism stabilizing cooperation, while partner control might be less frequent and limited to short-term interactions or to limited species that do not form stable social groups (*41*).

Why have we found such potent evidence of prosociality when previous studies failed to provide clear evidence? In their study of baboon communication, Seyfarth and Cheney (*42*) showed that it was essential to consider the social context in which the vocalizations occurred to understand the chacma baboons’ interpretation of the vocalization. Similarly, experiments on cooperation, as well as social cognition more broadly, are not realized in a “social vacuum” (*42*) but must be integrated into the history of interactions and relationships of individuals. Unfortunately, laboratory experiments often occur in a social vacuum, when the task is presented to individuals in an unfamiliar context in which they must be isolated, during a limited amount of time, and for a limited number of trials, thus limiting the chances of observing complex social strategies emerging (*22*, *31*). By contrast, more naturalistic experiments realized in a group context facilitate the emergence of complex social strategies, but crucial experimental controls are often limited in such a rich context. In comparison, through our unique seminaturalistic experimental setup, a group of 18 Guinea baboons has been participating voluntarily to weeks-long controlled experiments for years, making their participation for a few hours a part of their daily routine. We believe that this has profound consequences on the nature of the data that we can obtain and is the primary reason that we found these strong, clear results.

More generally, our results also invite a perspective on the evolutionary origins of human cooperation. Until now, the strongest levels of cooperation had been observed in cooperative breeding species, suggesting that cooperative breeding could be at the origin of human cooperation (*43*–*45*). Our results support a nonexclusive alternative hypothesis by showing that Guinea baboons, which are not cooperative breeders, can develop stable cooperation given a favorable environment allowing partner choice and partner control. Such an environment was likely typical in the evolution of human sociality and could have played a key role in favoring the development of nascent ancestral abilities.

## MATERIALS AND METHODS

### Prosociality in a seminatural cooperative setting

In our system, the baboons are free to come and go at any time (Fig. 1, A and B). When a baboon is identified by a computer, a task is presented on the touch screen, and if the baboon is successful, then a small reward of a few grains of puffed wheat is delivered (throughout the experiments, the reward is fixed; it does not vary in quality or quantity); otherwise, a green “time-out” screen appears for 3 s. To study the emergence of cooperation, we used one of the most frequently used tests in animal studies, the PCT (*46*). In the PCT, an individual must choose between two actions, one of which results in the delivery of a reward to themselves and their partner (prosocial choice) and the other that only rewards themselves (selfish choice). Despite a large research effort, results from PCT studies in NHPs are mixed (*24*–*26*, *34*) and suggest that giving monkeys more opportunities for strategic behavior could potentially lead to the establishment and stabilization of cooperation.

To implement the PCT, we used pairs of testing systems with transparent partitions that allowed two individuals to see each other, each other’s actions on the touchscreens, and each other’s reward or time-out screens. When two monkeys were identified in neighboring devices at the same time, they were randomly assigned the role of actor, who would have to make the choice, or the role of passive receiver. Note that for every trial, the role was assigned randomly by the computer, resulting in bouts of trials during which two individuals stayed with each other, alternating their role of actor and receiver (mean bout length during experiment 1: 4.2 trials, min = 1, max = 112). In the absence of a partner, baboons could still perform a solo task, thus avoiding the cooperative task (more details about the solo task in fig. S1). However, all the individuals performed significant amounts of trials with a partner, thus showing a voluntary participation in the cooperative task [during experiment 1, for instance, baboons performed 57% of cooperative task trials on average (SD = 15%, min = 32%, max = 97%)].

In experiment 1, the actor could choose between the “prosocial” stimulus, which rewarded the actor and the receiver (1-1), the “selfish” one, which rewarded only the actor (1-0), and the “control” one, which rewarded neither (0-0) (see fig. S2 for more details). If the actor chose the prosocial stimulus during this baseline phase, then this could be not only for social reasons (understanding that their choice would reward their partner) but also for nonsocial reasons (e.g., stimulus preference). To control for this last possibility, we reversed the valence of the two stimuli: The previously prosocial stimulus became the selfish one and vice versa (Fig. 1C). Truly prosocial baboons should adapt to this change and choose the new prosocial stimulus. Last, to control any other reason that might cause the monkeys to make the prosocial choice, we also performed a “ghost” control condition (*47*) following the same reversal protocol but with no receiver present (see Supplementary Materials and Methods for details).

### Ethics statement

This research was carried out in accordance with European Union and French ethical standards and received approval from the French Ministère de l’Education Nationale et de la Recherche (approval no. APAFIS-2717-2015111708173794-V3).

## Supplementary Materials

### This PDF file includes:

Materials and Methods Figs. S1 to S18 Tables S1 to S9 Legends for movies S1 to S5 References
