# Supplementary material for: Guinea baboons are strategic cooperators
Source: Sci Adv. 2023 Oct 27;9(43):eadi5282. doi: 10.1126/sciadv.adi5282 (PMC10610893; doi:10.1126/sciadv.adi5282)
Supplement: Supplementary file 1 — Materials and Methods Figs. S1 to S18 Tables S1 to S9 Legends for movies S1 to S5 References [file sciadv.adi5282_sm.pdf]

Science Advances
AAAS

Supplementary Materials for

Guinea baboons are strategic cooperators

Anthony Formaux *et al.*

Corresponding author: Nicolas Claidière, nicolas.claidiere@cnrs.fr

Sci. Adv. **9**, eadi5282 (2023)
DOI: 10.1126/sciadv.adi5282

The PDF file includes:

Materials and Methods
Figs. S1 to S18
Tables S1 to S9
Legends for movies S1 to S5
References

Other Supplementary Material for this manuscript includes the following:

Movies S1 to S5

Material and methods

Participants

Eighteen Guinea baboons (*Papio papio*, twelve females, Table S1) from the CNRS primatology centre in Rousset-sur-Arc participated in this study (median age 12 years old, min = 5, max = 24). They belonged to a social group living in an enriched outdoor enclosure of 25x30m connected to an indoor enclosure of 6x4m, and two experimental trailers (8x4m) with 2 and 3 s-ALDM (social automated learning device for monkeys, see below) respectively.

Ethics:

This research was carried out in accordance with European Union and French ethical standards and received approval from the French Ministère de l'Education Nationale et de la Recherche (approval no. APAFIS-2717-2015111708173794-V3).

Name	Sex	Age (Month)
ANGELE	F	200
ARIELLE	F	195
**ATMOSPHERE**	**F**	**286**
BOBO	M	185
EWINE	F	150
FANA	F	143
FELIPE	M	140
**FEYA**	**F**	**137**
HARLEM	M	114
**LIPS**	**F**	**74**
**LOME**	**M**	**77**
**MAKO**	**M**	**66**
**MALI**	**F**	**71**
**MUSE**	**F**	**67**
NEKKE	F	51
PETOULETTE	F	274
PIPO	M	269
**VIOLETTE**	**F**	**205**

Table S1: Characteristics of individuals participating in the study. In bold, prosocial individuals.

Social Automated Learning Devices for Monkeys (S-ALDM)

ALDMs (*44, 45*) are fully automatic operant conditioning test systems that can be used for testing non-human primates in social settings without the need to capture or isolate them. They use an automatic radio frequency identification device (RFID) implanted in each forearm of the monkeys to gather information about the location, identity, and current task of specific individuals. S-ALDMs are a modified version of the original ALDM developed by J.F. S-ALDMs are pairs of ALDMs that can allow a reciprocal visual access between two individuals and their screen (*46*). This is achieved using a transparent partition when visual access is required, or an opaque partition when not. In ALDMs tasks, a correct choice results in the delivery of a reward (grains of wheat), accompanied with a black screen. An incorrect choice does not result in the delivery of a reward and a 3s time out green screen is displayed. Importantly, when two participants participate in a S-ALDM task with a transparent

partition they can see the respective outcome of their trials because they can see the black screen, hear the delivery of the reward, and see they partner eating when they are successful, and they can see the green screen when they are not.

The operating principles of the S-ALDMs are as follows. When one monkey is identified a blue screen appears for a maximum of four seconds. During the delay, if another individual is identified in the neighboring S-ALDM, a “dual task” is launched for both (Tab. S1). If there is no identification of a partner in the neighboring S-ALDM during the four seconds delay, a “filler task” starts (Fig. S1).

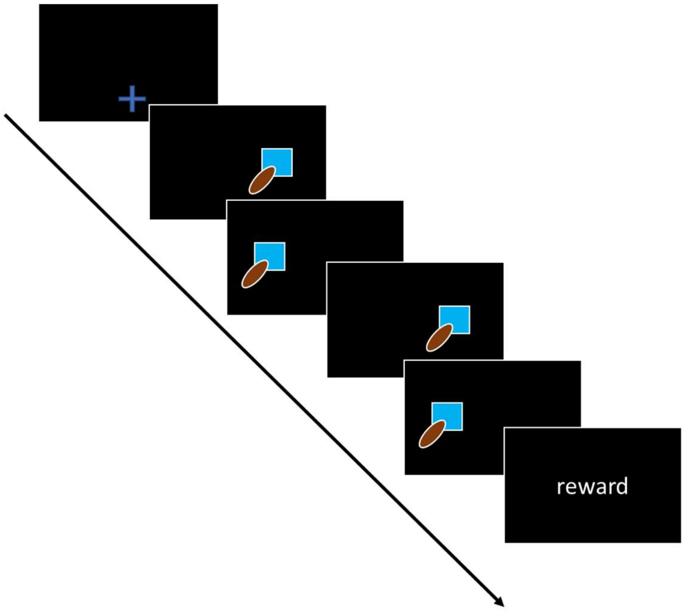

Figure S1: Filler task used in the experiments. The filler task we used was an adaptation of the Fitts task (*48*) that is used to measure the time required to go from one point to another. Baboons had to touch a square appearing successively on the right and left of the screen. At the start of a trial, a square first appeared on one side of the screen, when touched, it disappeared, and re-appeared on the opFirposite side of the screen. This was repeated twice so that monkeys had to touch the square four times in alternating position. If successful, baboons were rewarded but if a square was missed, the trial stopped and was considered a failure and a green screen was displayed for 3 secs, no reward was

provided. The function of the filler task was to let the baboons use the S-ALDM in the absence of another individual. The data from the filler task were not analyzed.

Data analysis

Due to technical errors 0.34% of trials happened with a misidentification of the partner (noted “IdError” in the data). We performed all analyses reported below with and without these trials and found no substantial difference. Since the misidentification happened for the partner and we still know the choice of the focal individual we decided to report the results based on the full set of trials to maintain the balanced number of trials between conditions. All the data and analysis code are available to reproduce the analyses and figures below. See https://osf.io/dmujs/

Experiment 1 : test condition

During the dual task, a fixation cross appeared on each screen. The roles of the two individuals were then randomly chosen by the test program for each trial. The individual selected as the actor had to choose among three images randomly predefined as the prosocial choice, the selfish choice, and the control choice. The positions of the images were randomized for each trial to the top, middle, or bottom positions. The other individual, the receiver, was in a waiting position with a black screen. As soon as the actor made their choice, the outcome of the trial was determined first for the receiver, then, 1500ms later, for the actor. This delay was introduced to give the actor time to see the consequences of their choice on the receiver, and to avoid focusing exclusively on their outcome (reward or time out). Prosocial and selfish trials are illustrated in Figure S2. When the actor selected the control stimulus, the partner screen displayed a green screen, followed by a green screen for the actor and none of them received food rewards.

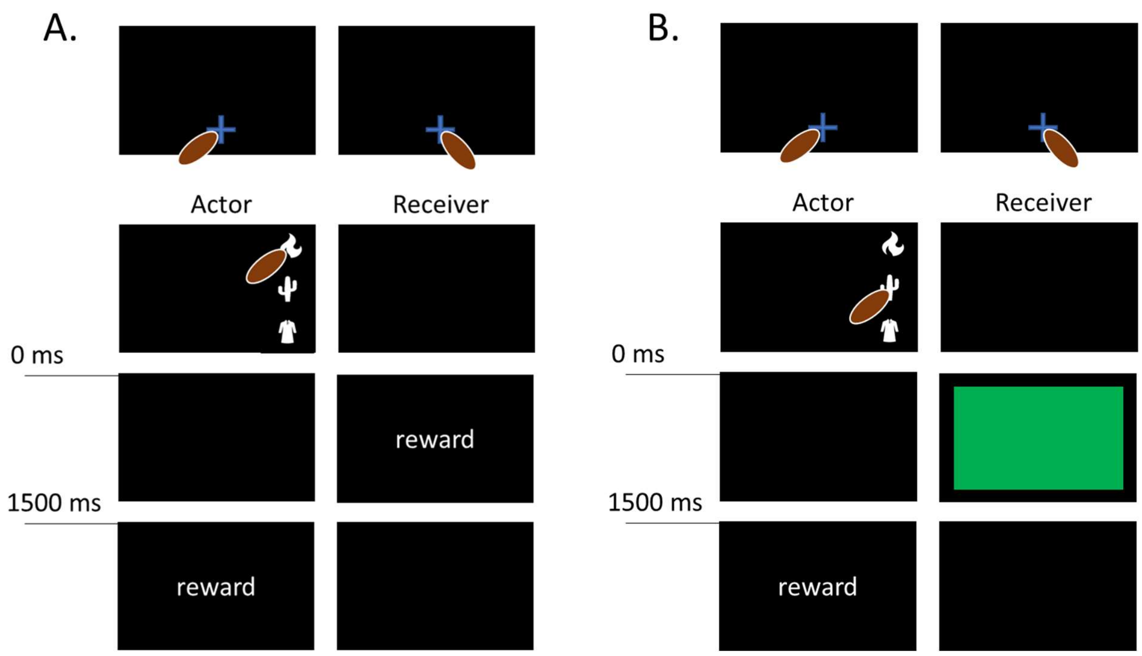

Figure S2: Prosocial and selfish trials. A: The actor makes the prosocial choice. The receiver is rewarded, and a black screen appears on the actor's side. In parallel, 1500ms after the beginning of the actor choice, a reward is delivered to the actor's side. B: Selfish trial. The actor makes the selfish choice, and a black screen appears on the actor's side. The receiver is not rewarded, and a green screen appears on their side. In parallel, 1500ms after the actor choice, a reward is delivered to the actor's side.

The test condition started with a baseline phase in which the stimuli were presented and followed by a reverse phase during which the valence of the prosocial and selfish stimuli was reversed. Our prediction was that if monkeys are prosocial, they should choose the prosocial stimuli in both the baseline and reverse condition. As in previous experiments, we define a threshold of 80% prosocial choice in a set of 50 trials as the criterion to determine a change (e.g. *46, 47*).

<u>Experiment 1 : Ghost control condition</u>

During the ghost control condition, we closed one of the two access to the S-ALDM, so that only one monkey could use the S-ALDM, with no partner present. When a trial started, the individual was automatically selected as an actor, and the 'receiver' was simulated by the computer. The trial

continued as in the test phase: the fixation cross appeared, followed by three different images with the same outcomes (prosocial, selfish, and control). The ghost control condition also started with a baseline phase, followed by the reverse condition. The main objective of this phase was to establish the probability that monkeys would choose the prosocial option in both baseline and reverse condition in the absence of a partner. This could have happened for the following reasons:

- The green screen appearing on the adjacent screen during a selfish choice could be a negative clue for the actor and be avoided, since they have been accustomed to the association of the green screen with a negative outcome (*49*).
- The sound of grain falling into the adjacent feeder might present a positive reinforcement, since monkeys are used to hearing the sound when they are rewarded.
- Finally, other unknown factors could have influenced the selection of the different stimuli.

Note that during the ghost phase, the 'ghost' S-ALDM delivered rewards but to avoid an accumulation of rewards in the 'ghost' S-ALDM we redirected the rewards into an opaque container.

In contrast to the experimental phase, our prediction was that monkeys would not change their response between the baseline and reverse conditions and therefore not choose the prosocial response in both conditions.

<u>Results</u>

Figure S3 and S4 show the results of the test and control condition for the baseline and reverse phase for all individuals. During the control condition, no baboons adopted a prosocial response in both the baseline and reverse phase, whereas eight did during the test phase (a significant difference with the control condition: binomial test, 0/18 vs. 8/18, $p < 0.001$). In the test condition, however, the number of trials is larger than the control condition. When we limit the analysis to the same number of complete blocks of 50 trials done by the same individual in the reverse phase of the test and control condition, 5 individuals passed our 80% criterion before reaching the number of blocks done in the

reverse control condition, still showing a strong significant difference (binomial test, 0/18 vs. 5/18, p <0.001).

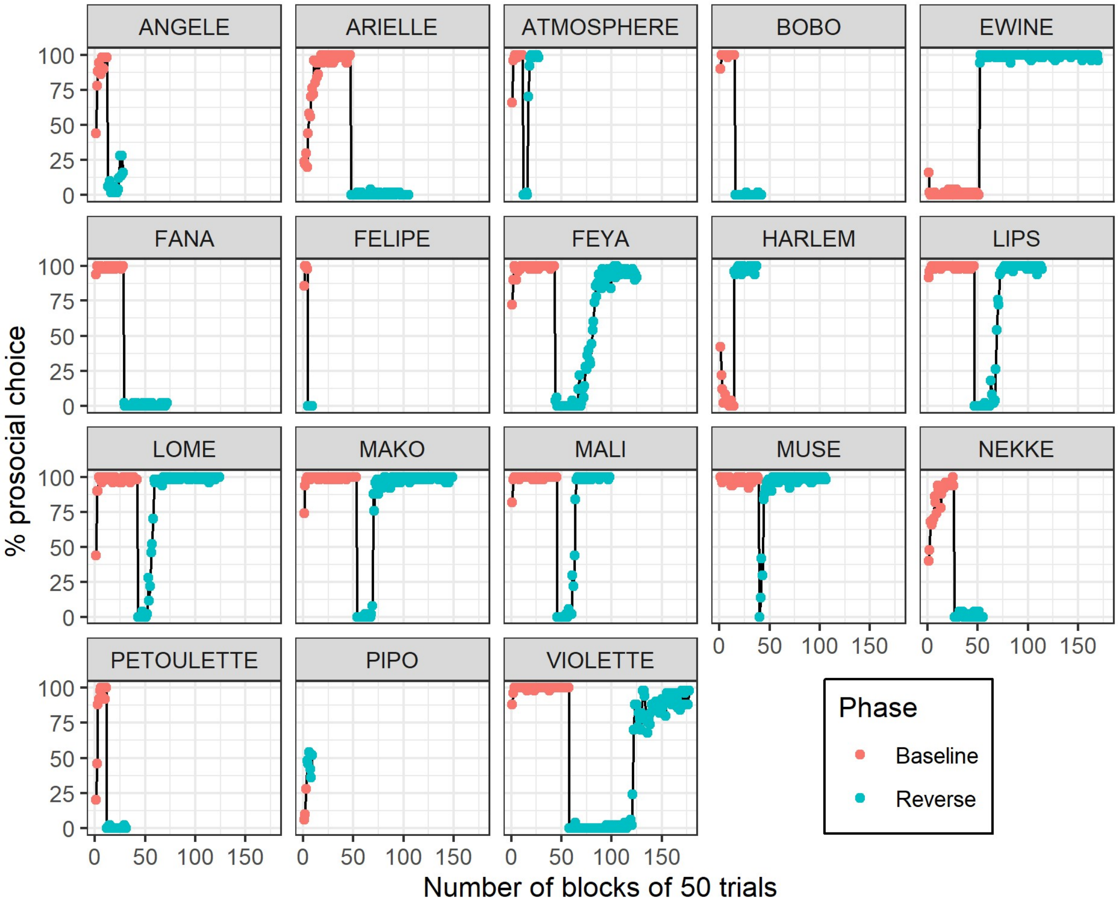

Figure S3: Proportion of prosocial choices in each block of 50 trials during the test condition.

Baseline phase (red) and reversal phase (blue).

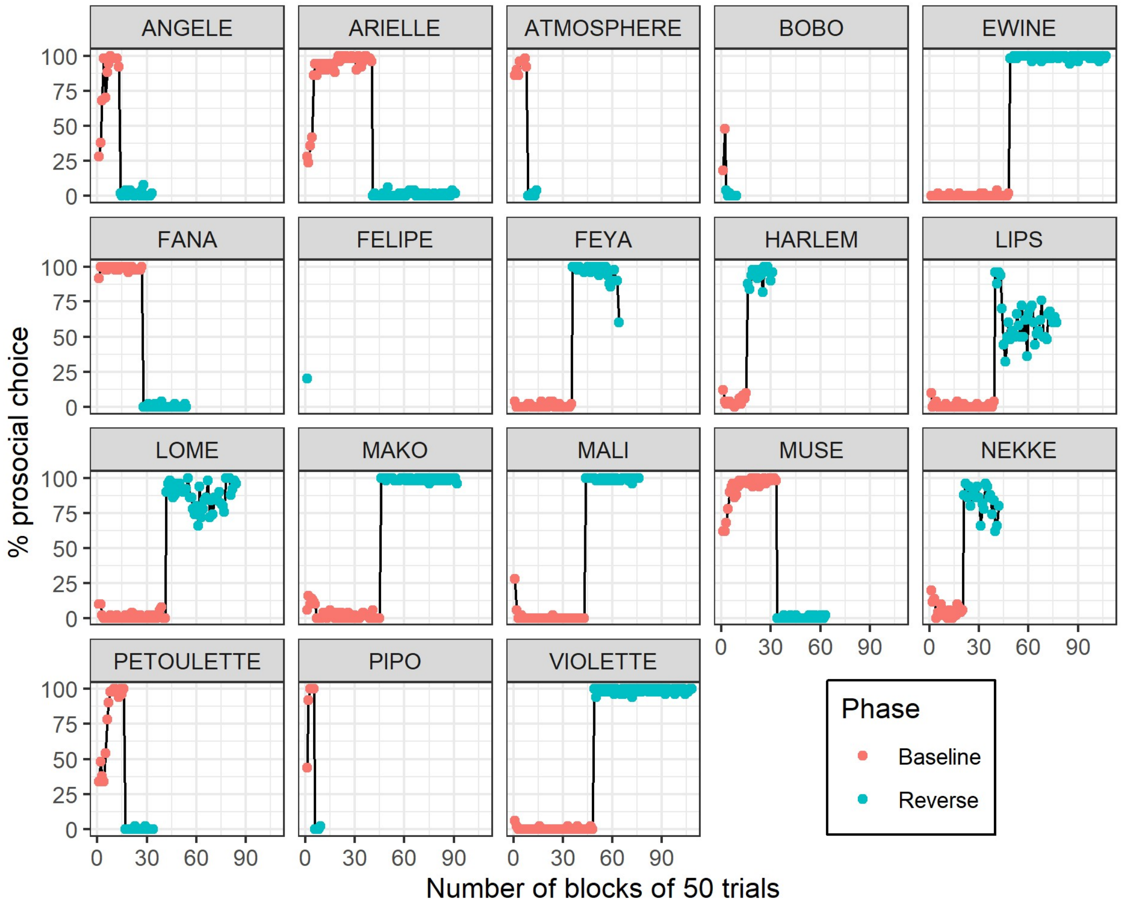

Figure S4: Proportion of prosocial choices in each block of 50 trials during the control condition.

Baseline phase (red) and reversal phase (blue).

<u>Experiment 2 : Training</u>

During experiment 2, we wanted to challenge their capacity to maintain cooperation by gradually introducing non rewarded PCT trials (NR-PCT) with one stimulus rewarding only the receiver (0-1), and the other giving no rewards (0-0;see main text). However, before starting Experiment 2, we wanted to make sure that the monkeys would not choose the prosocial stimulus by default at the start of the experiment. To remain conservative, we therefore decided to train them to choose the stimuli that would correspond to the selfish condition rather than the one used for the prosocial response. In this training phase, the two stimuli were presented in a forced choice task. Baboons were rewarded if they

chose the stimuli that would later become the selfish one and not rewarded if they chose the stimuli that later became the prosocial one.

Experiment 2: Testing

During testing we progressively introduced NR-PCT trials to the rewarded PCT trials (R-PCT) to give monkeys time to adapt to the new non-reinforced trials. Every two days, the proportion of NR-PCT trials increased, from 0% to 100% (see main text). Crucially, at the end of testing, only NR-pct trials remained and actors could therefore no longer receive rewards directly.

Results : Training

For the training phase, the eighteen participating individuals all reached 80% of success in a block of 50 trials (mean number of blocks to reach the criterion: 1.2, min = 1, max =2).

Results : Testing

We analyzed separately blocks of 50 NR-PCT trials and of 50 R-PCT trials for the 8 prosocial monkeys revealed during experiment 1 (Fig S5). We found that all reached our criterion of 80% prosocial choice on a block of 50 trials at least once in each condition.

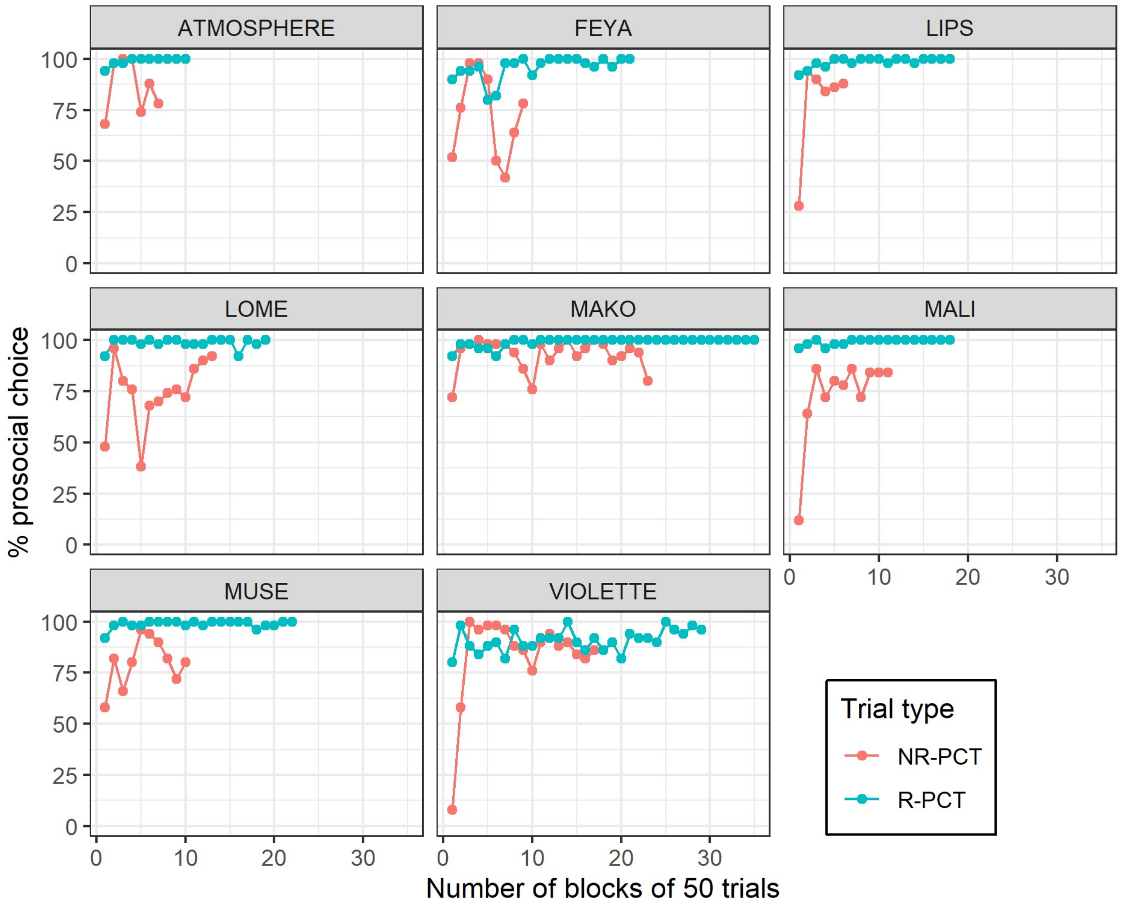

Figure S5: Results of the test phase of experiment 2 for prosocial monkeys. Proportion of prosocial choices in each complete block of 50 NR/R-PCT trials.

<u>Analysis of behavioral strategies : Reciprocity</u>

We examined the probability that a monkey chose the prosocial stimuli depending on their partner's previous response. To do that we selected all cases in which partners were the same but the roles were exchanged between two successive trials performed in less than 15 secs apart. For experiment 1, we selected trials that were done after the baboons had reached 80% prosocial choice and given the high level of prosocial choice, we found no evidence of reciprocity, the eight prosocial individuals made their choice regardless of their partner's previous behavior (Fig S6, Tab S2).

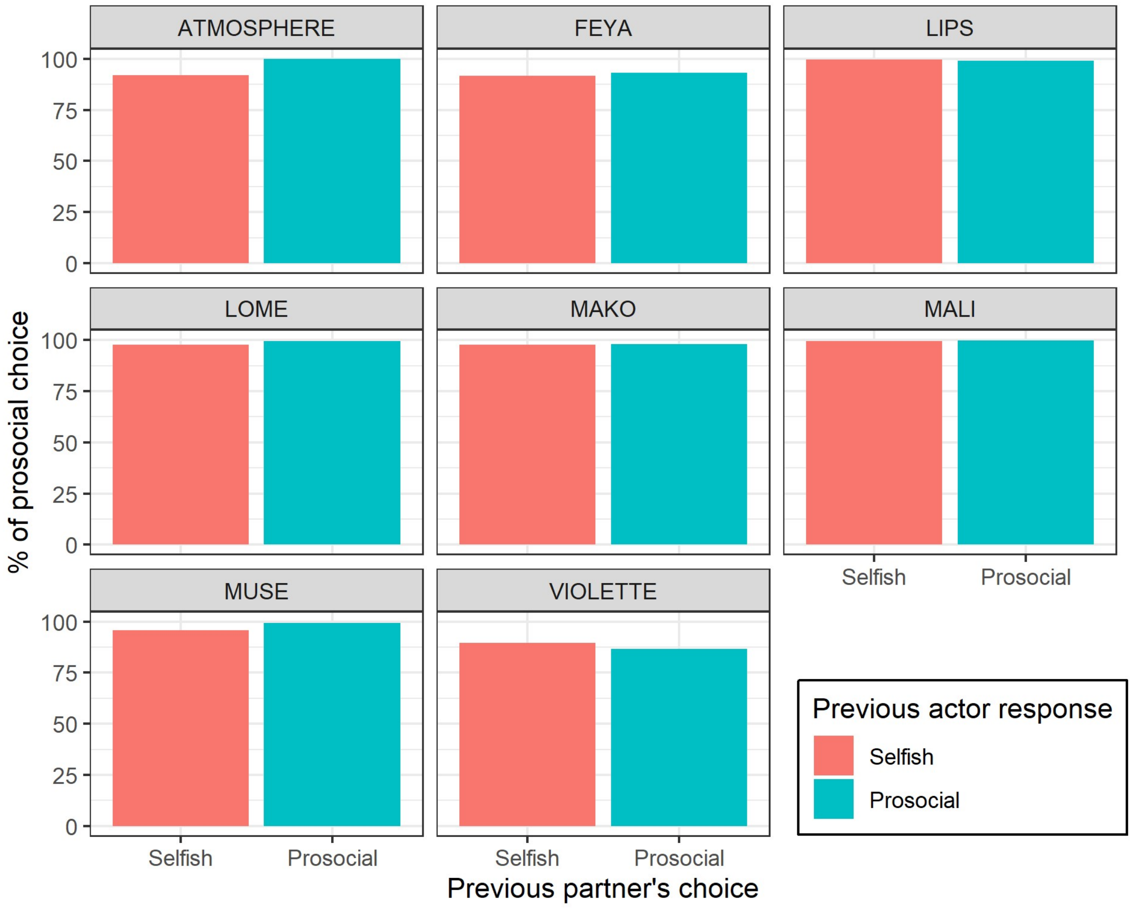

Figure S6: Individual results for the eight prosocial individuals during experiment 1. Proportion of prosocial choice after receiving a reward or not.

**Random effect**	**Variance**	**Std.Dev.**		
(Intercept)	1.32	1.15		
**Fixed effect**	**Estimate**	**Std.Error**	**z value**	**Pr(>|z|)**
(Intercept)	3.75	0.44	8.53	<0.001
Prosocial	0.28	0.15	1.83	0.07

Table S2: Analysis of reciprocity for experiment 1. GLMM results of the binomial model including as dependent variable the binary choice of the actor (Selfish = 0, Prosocial = 1), depending on the choice of the partner in the previous trial (Selfish or Prosocial) and including a random intercept for the actor, accounting for repeated measures. GLMM analysis was performed using R lme4 package (*50*).

Regarding experiment 2, we found that for NR-PCT trials, all 8 individuals were more likely to choose the prosocial response after their partner had done the same compared to when the partner had chosen the selfish response (Fig S7, Tab S3).

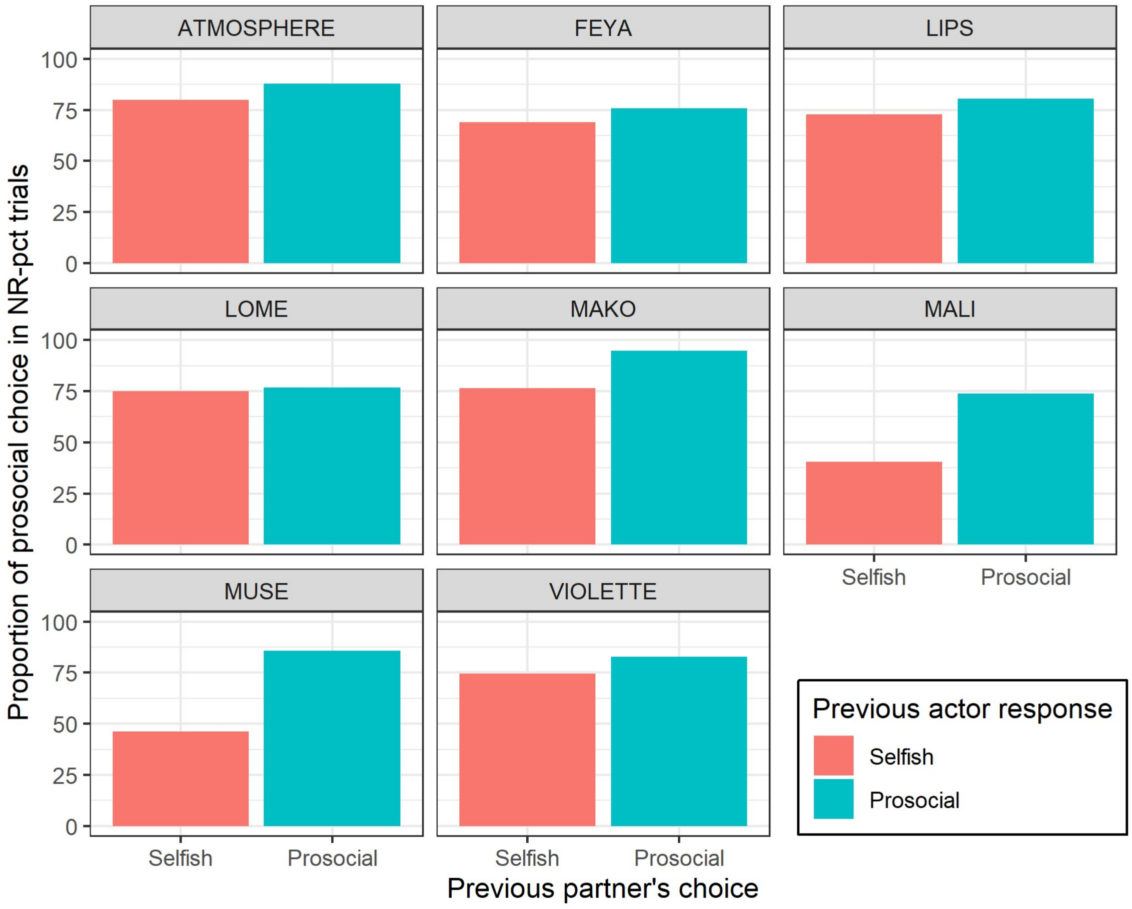

Figure S7: Individual results for the eight prosocial individuals during experiment 2. Proportion of prosocial choice after receiving a reward or not for NR-PCT trials.

Random effect	Variance	Std.Dev.		
(Intercept)	0.21	0.46		
**Fixed effect**	**Estimate**	**Std.Error**	**z value**	**Pr(>|z|)**
(Intercept)	0.71	0.21	3.30	<0.001
Prosocial	0.91	0.16	5.81	<0.001

Table S3: Analysis of reciprocity for experiment 2. GLMM results of the binomial model including as dependent variable the binary choice of the actor (Selfish = 0, Prosocial = 1), depending on the choice of the partner in the previous trial (Selfish or Prosocial) and including a random intercept for the actor, accounting for repeated measures. GLMM analysis was performed using R lme4 package (*50)*.

<u>Analysis of behavioral strategies : partner choice</u>

In addition, we found that in both experiments prosocial monkeys were more likely than non-prosocial monkeys to change partner when their partner chose the selfish stimuli in the previous trial (Fig. S8). This form of partner choice existed in both prosocial and non-prosocial monkeys but was significantly stronger in the former (Tab S4).

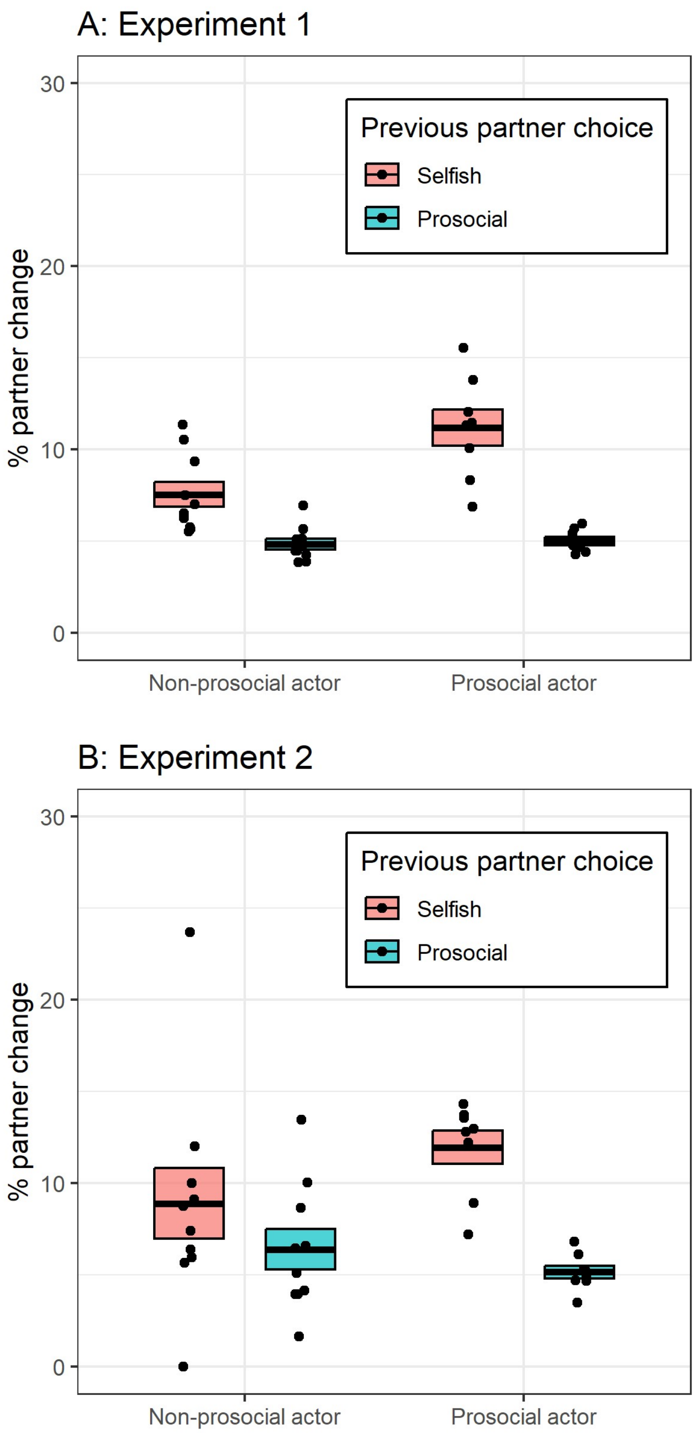

Figure S8: Proportion of trials in which partners change when the prosocial or non-prosocial actor was a receiver in the previous trial and received either a prosocial outcome or a selfish one. Panel A, experiment 1 (R-PCT) and panel B, experiment 2 (NR-PCT).

Experiment 1

<table>
<tr><td>Random effect</td><td>Variance</td><td>Std.Dev.</td><td></td><td></td></tr>
<tr><td>(Intercept)</td><td>0.018</td><td>0.13</td><td></td><td></td></tr>
<tr><td>Fixed effect</td><td>Estimate</td><td>Std.Error</td><td>z value</td><td>Pr(>|z|)</td></tr>
<tr><td>(Intercept)</td><td>-2.48</td><td>0.06</td><td>-40.9</td><td><0.001</td></tr>
<tr><td>Previous response :
Prosocial</td><td>-0.53</td><td>0.05</td><td>-9.82</td><td><0.001</td></tr>
<tr><td>Actor : Prosocial</td><td>0.38</td><td>0.08</td><td>4.61</td><td><0.001</td></tr>
<tr><td>Interaction</td><td>-0.34</td><td>0.07</td><td>-5.07</td><td><0.001</td></tr>
</table>

Experiment 2

<table>
<tr><td>Random effect</td><td>Variance</td><td>Std.Dev.</td><td></td><td></td></tr>
<tr><td>(Intercept)</td><td>0.075</td><td>0.27</td><td></td><td></td></tr>
<tr><td>Fixed effect</td><td>Estimate</td><td>Std.Error</td><td>z value</td><td>Pr(>|z|)</td></tr>
<tr><td>(Intercept)</td><td>-2.27</td><td>0.12</td><td>-18.5</td><td><0.001</td></tr>
<tr><td>Previous response :
Prosocial</td><td>-0.40</td><td>0.09</td><td>-4.56</td><td><0.001</td></tr>
<tr><td>Actor : Prosocial</td><td>0.27</td><td>0.17</td><td>1.58</td><td>0.11</td></tr>
<tr><td>Interaction</td><td>-0.54</td><td>0.12</td><td>-4.59</td><td><0.001</td></tr>
</table>

Table S4: Results of the analysis of partner change in experiment 1 and 2. GLMM results of the binomial model including as dependent variable whether or not the trial previous trial was with the same partners (Same = 0, Change = 1), depending on the choice of the partner in the previous trial (Selfish or Prosocial) and with an interaction depending on whether the actor is part of the group of prosocial monkeys or not. The model also included a random intercept for the actor, accounting for repeated measures. GLMM analysis was performed using R lme4 package (*50*).

This partner choice created a situation in which there was a positive correlation between the number of trials performed by a pair of individuals and their joint level of prosociality (Fig S9).

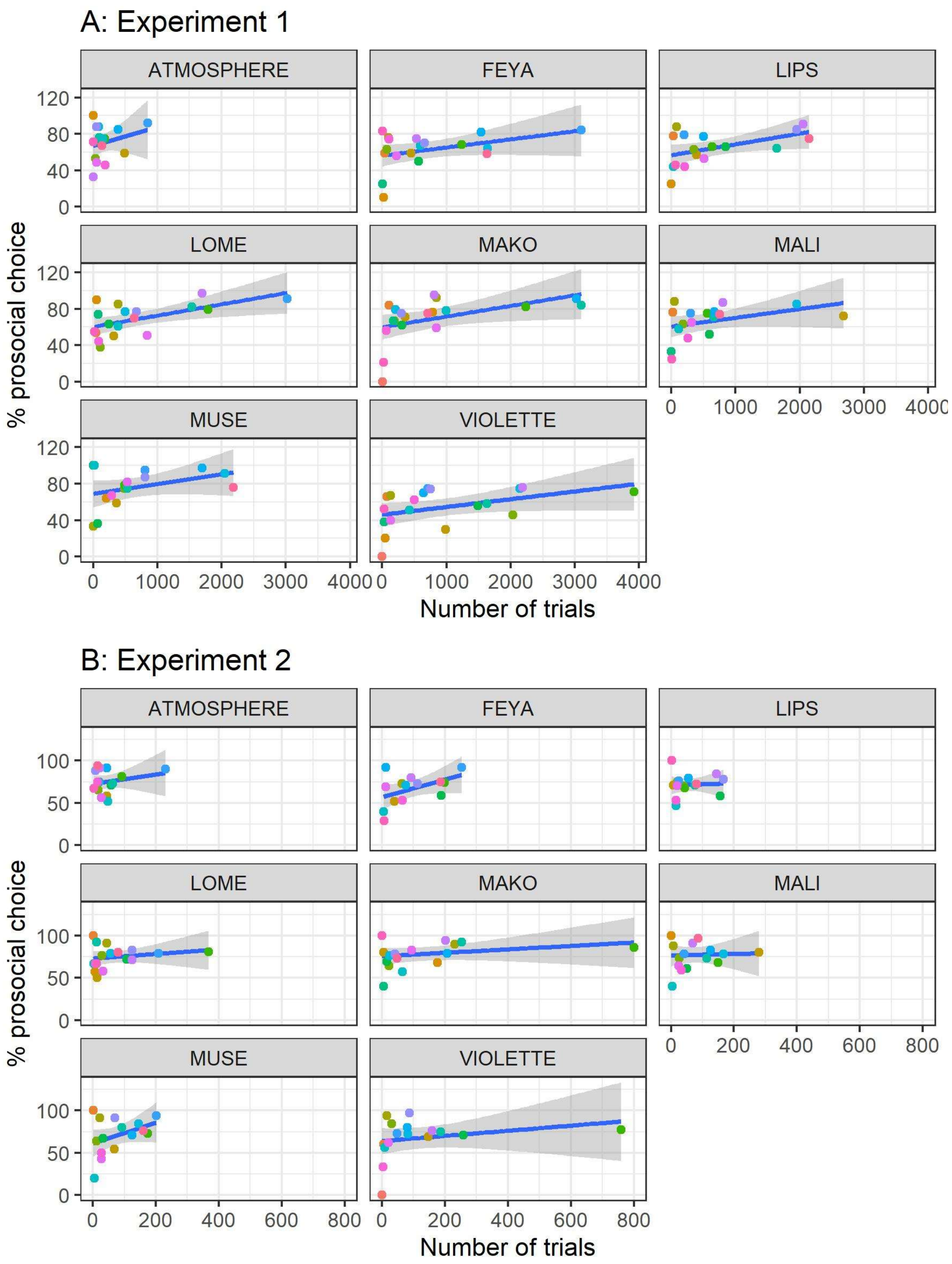

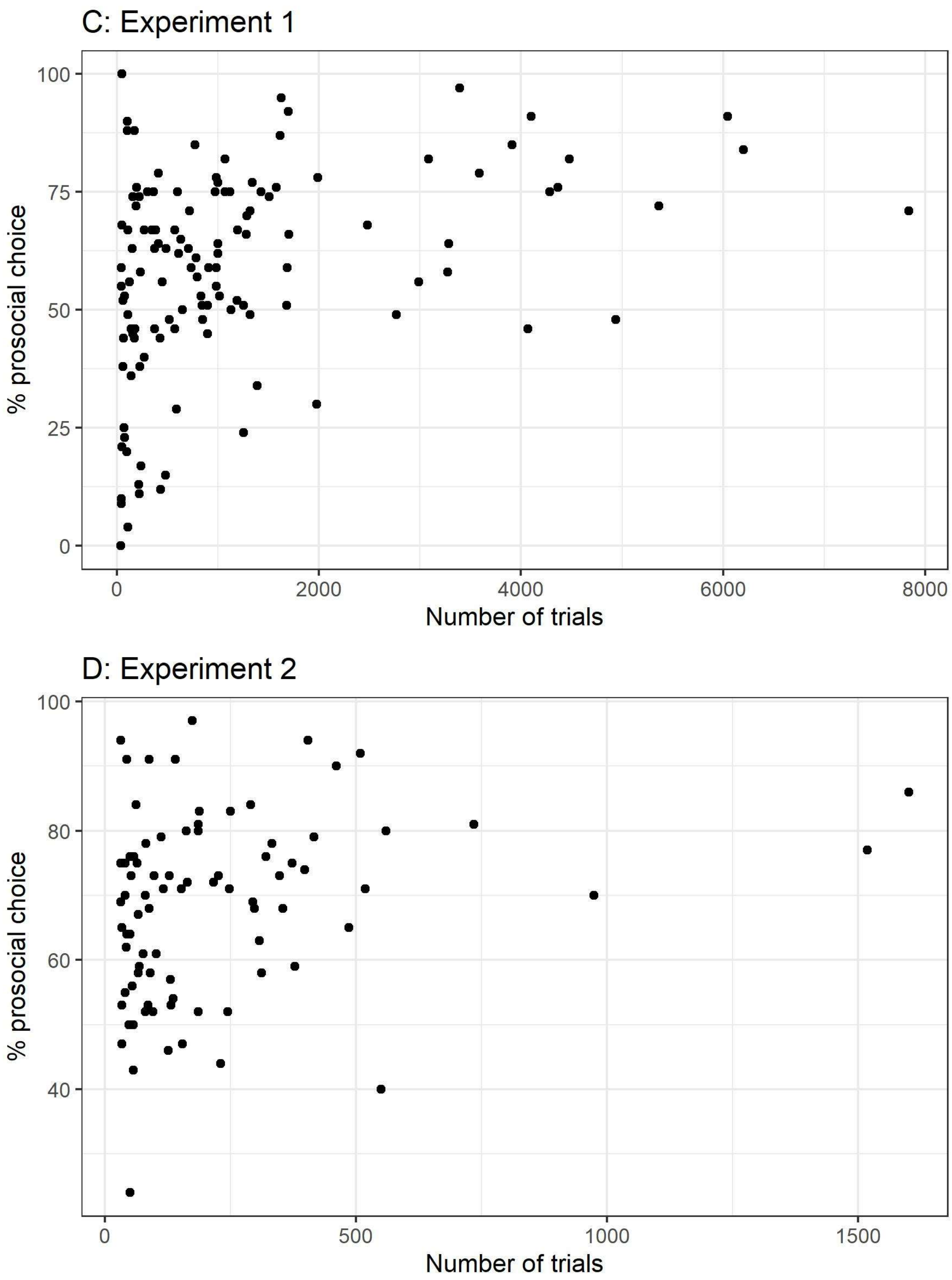

Figure S9: Correlation between the proportion of prosocial choice between pairs of individuals and the number of trials that the pair has done. **A:** Individual correlation for prosocial monkeys during experiment 1 (R-PCT). B: Individual correlation for prosocial monkeys during experiment 2 (NR-PCT). **C:** Group correlation for every pair (prosocial and non-prosocial) with at least 30 trials during

experiment 1 (R-PCT). **D:** Group correlation for every pair (prosocial and non-prosocial) with at least 30 trials during experiment 2 (NR-PCT).

We found a reliable positive relationship for prosocial monkeys (Fig.S9 A & B). Since the data are non-independent, we used a non-parametric Spearman test that showed a positive relationship between the number of trials and the proportion of prosocial choice for pairs of individuals (Fig. S9 C & D; Experiment 1, R-PCT trials, Spearman rho = 0.31, $p<0.001$; Experiment 2, NR-PCT trials, Spearman rho = 0.32, $p<0.001$).

<u>Analysis of behavioral strategies: Interrupted trial strategy</u>

During the experiment, if a response was not obtained within a certain duration, the trial was considered as 'interrupted' and terminated (without delivery of a reward or presence of a time out). There were two moments during which a trial could be interrupted (Fig S10):

- when there was no response during the fixation cross, possible for both the actor and the receiver

- when there was no response during the choice screen, possible for the actor only

We noticed that the rate of interrupted trials almost doubled between the two experiments for prosocial monkeys (7 % [min = 5%, max = 9%] of interrupted trial in experiment 1 and 13% [min = 5%, max = 22%] in experiment 2).

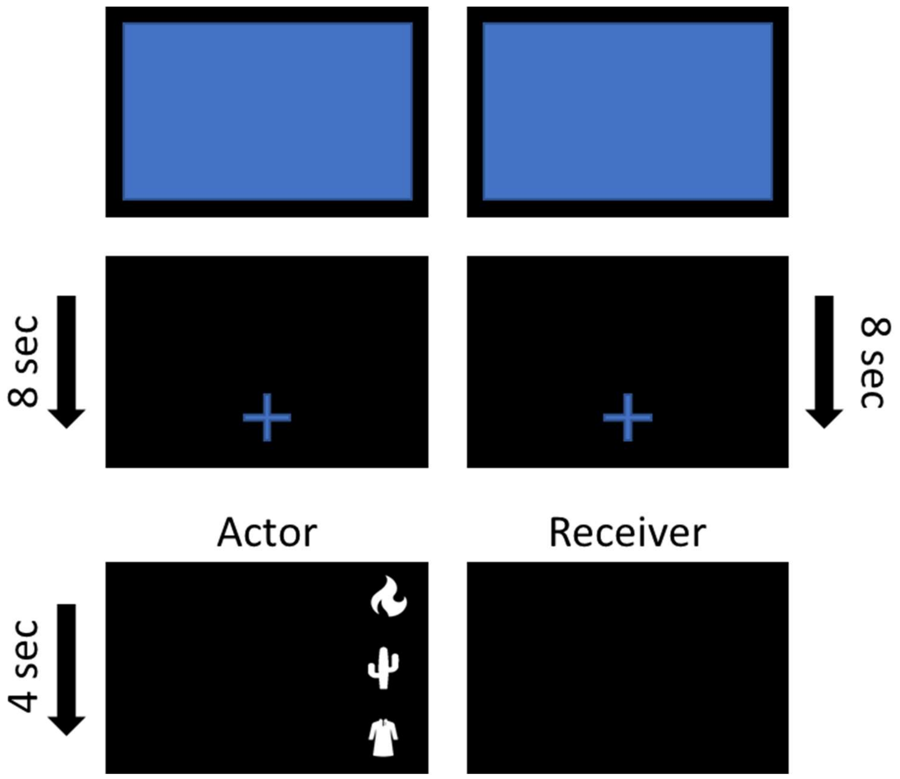

Fig S10: Interrupted trials. When there was no response within 8 secs during the fixation cross or within 4 secs during the choice screen, the trial was considered as 'interrupted', and there was no feedback, the program re-started from the beginning.

We compared the proportion of interrupted trials between the fixation cross and the choice screen, for prosocial and non-prosocial individuals (Fig. S11). This is close to 50% for non-prosocial individuals and slightly larger for prosocial individuals during experiment 1 (Tab. S5). We find similar results during experiment 2, except that the proportion of interrupted trials during the choice screen increased sharply for prosocial monkeys when they were in the role of actor (from a mean = 0.62 [SE = 0.04] to mean = 0.82 [SE = 0.05]).

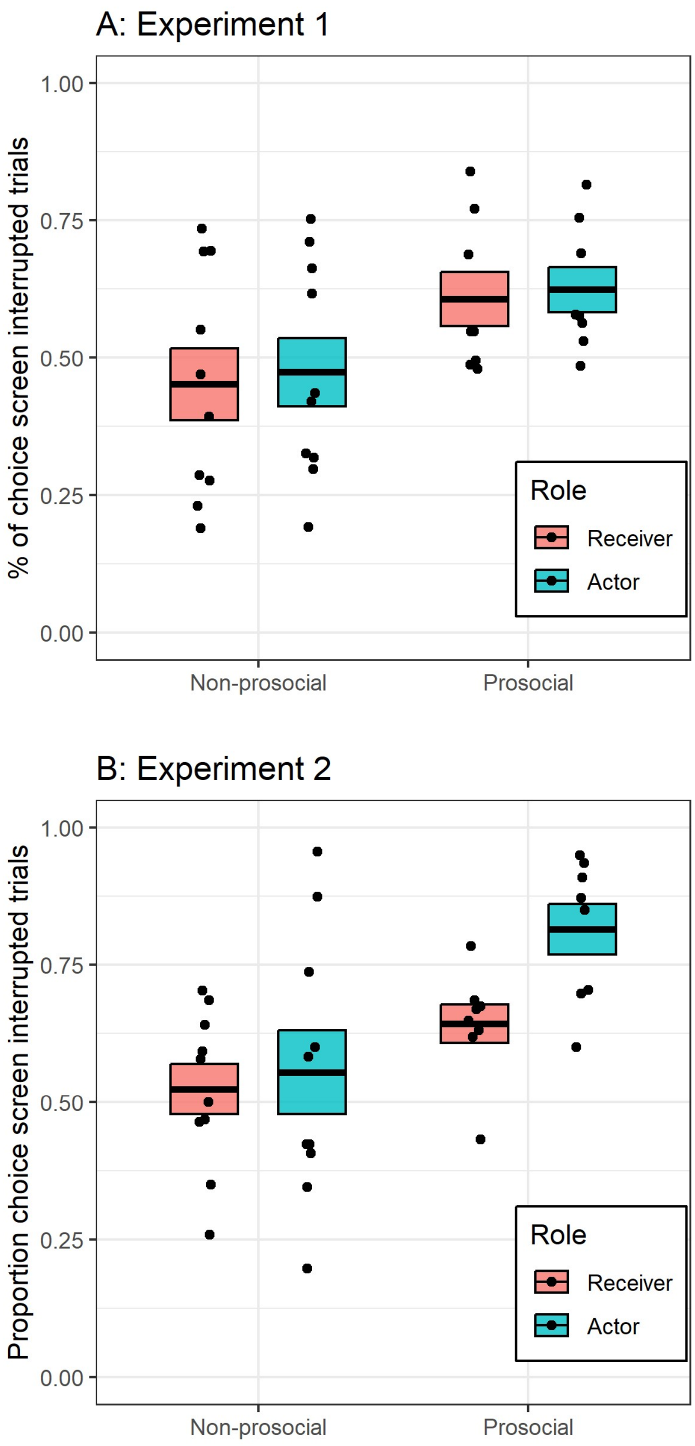

Figure S11: Proportion of interrupted trial during the choice screen with a prosocial or non-prosocial individual depending on their role. We found a clear increase in the abortion rate of prosocial monkeys when they were actors during the second experiment. All R-PCT and NR-PCT trials are included.

Experiment 1

Random effect	Variance	Std.Dev.
(Intercept)	0.52	0.72

Fixed effect	Estimate	Std.Error	z value	Pr(>|z|)
(Intercept)	-0.22	0.23	-0.95	0.34
Role: Actor	0.10	0.06	1.74	0.08
Actor : Prosocial	0.70	0.35	2.01	0.04
Interaction	-0.03	0.08	-0.42	0.68

Experiment 2

Random effect	Variance	Std.Dev.
(Intercept)	0.075	0.27

Fixed effect	Estimate	Std.Error	z value	Pr(>|z|)
(Intercept)	-0.12	0.27	-0.45	0.65
Role: Actor	0.75	0.10	7.28	<0.001
Actor : Prosocial	0.68	0.39	1.73	0.08
Interaction	0.54	0.14	3.83	<0.001

Table S5: Results of the interrupted trials analysis. GLMM results of the binomial model including as dependent variable whether or not the trial was interrupted during the choice screen (No = 0, Yes = 1), depending on the role of the individual (Receiver or Actor) and with an interaction depending on whether the actor is part of the group of prosocial monkeys or not. The model also included a random intercept for the actor, accounting for repeated measures. GLMM analysis was performed using R lme4 package (*50*).

This increase in interrupted trials specific to prosocial individuals and to the choice screen shows that prosocial individuals changed their strategy. Given that monkeys changed partners more often after an interrupted trial (Fig. S12 and Tab S6) and were more likely to abort a trial when the partner had previously chosen a selfish response (Fig. S13 and Tab S7), this shows another form of reciprocity and partner choice: when their partner did not make the prosocial choice, prosocial monkeys were more likely to not respond when their turn came (i.e. interrupted) and change partner.

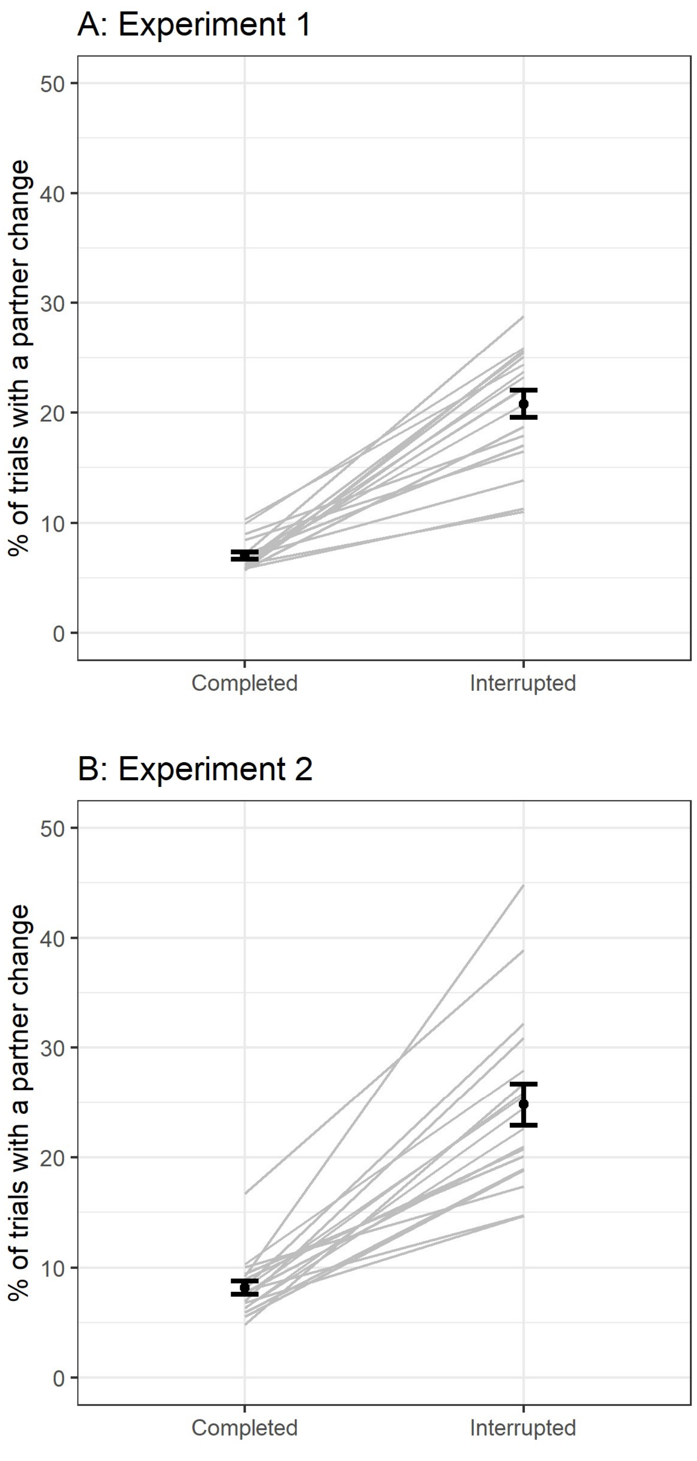

Figure S12: Proportion of trials with a change in partners after a completed or interrupted trial. Grey lines indicate individual results. All R-PCT and NR-PCT trials are included.

Experiment 1

Random effect	Variance	Std.Dev.		
(Intercept)	0.04	0.19		
**Fixed effect**	**Estimate**	**Std.Error**	**z value**	**Pr(>|z|)**
(Intercept)	-2.65	0.05	-57.3	< 0.001
Previous trial : Interrupted	1.32	0.03	52.5	< 0.001

Experiment 2

Random effect	Variance	Std.Dev.		
(Intercept)	0.072	0.27		
**Fixed effect**	**Estimate**	**Std.Error**	**z value**	**Pr(>|z|)**
(Intercept)	-2.44	0.07	-36.3	< 0.001
Previous trial : Interrupted	1.15	0.03	33.3	< 0.001

Table S6: Analysis of the probability to change partner after a completed or interrupted trial. GLMM results of the binomial model including as dependent variable the binary partner change (No change = 0, Change = 1), depending on the previous trial interrupted status (Completed or Interrupted) and including a random intercept for the actor, accounting for repeated measures. GLMM analysis was performed using R lme4 package (*50*).

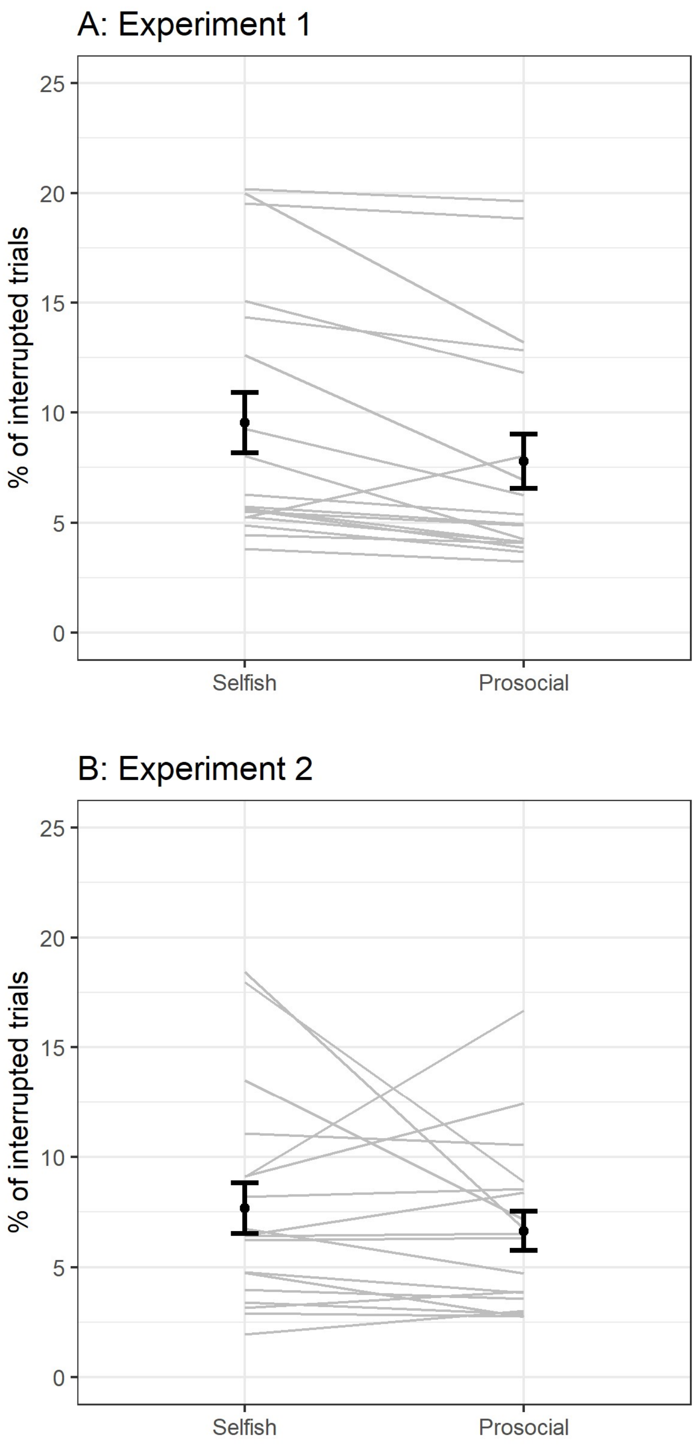

Figure S13: Proportion of trials with the same partners that are interrupted after a prosocial or selfish response (for all trials that are less than 15 secs apart). Grey lines indicate individual results. All R-PCT and NR-PCT trials are included.

Experiment 1

**Random effect**	**Variance**	**Std.Dev.**		
(Intercept)	0.37	0.61		
**Fixed effect**	**Estimate**	**Std.Error**	**z value**	**Pr(>|z|)**
(Intercept)	-2.66	0.13	-19.8	< 0.001
Previous trial : Selfish	0.26	0.03	10.0	< 0.001

Experiment 2

**Random effect**	**Variance**	**Std.Dev.**		
(Intercept)	0.27	0.52		
**Fixed effect**	**Estimate**	**Std.Error**	**z value**	**Pr(>|z|)**
(Intercept)	-2.84	0.13	-22.1	< 0.001
Previous trial : Selfish	0.17	0.05	3.10	< 0.001

Table S7: Analysis of the probability to interrupt a trial depending on the partner previous behaviour. GLMM results of the binomial model including as dependent variable the binary interrupted trial variable (Completed = 0, Interrupted = 1), depending on the choice of the partner in the previous trial (Selfish or Prosocial) and including a random intercept for the actor, accounting for repeated measures. GLMM analysis was performed using R lme4 package (*50*).

<u>Experiment 3 : Preliminary non-social task.</u>

During experiment 3, we wanted to explore the possibility that baboons would be willing to pay a small cost to maintain cooperation. Our first aim was to verify that they would differentiate a more costly choice to a less costly one, and that they would choose the former preferentially. We started a nonsocial task (Fig. S16) in which baboons were separated by an opaque partition from their partner. When a trial started, they could choose between three different stimuli. The control stimulus delivered

no reward and a 3s time out green screen was displayed. The non-costly stimulus delivered a reward. The costly stimulus had to be touched twice with a variable delay between the two touches to deliver a reward (the position of the stimulus randomly changed between the two touches). We manipulated the delay of the costly stimulus, starting with a 5000ms delay, so a monkey had to wait 5000ms between the first touch and the second touch, whereas choosing the non-costly choice would be immediately rewarding. We then progressively decreased the delay to determine when they would not avoid the costly stimuli. The following delay were used in that order: 5000ms, 4000ms, 3000ms, 2000ms, 1000ms, 500ms, 50ms. To obtain a reliable estimate, for each delay we used six different pair of stimuli.

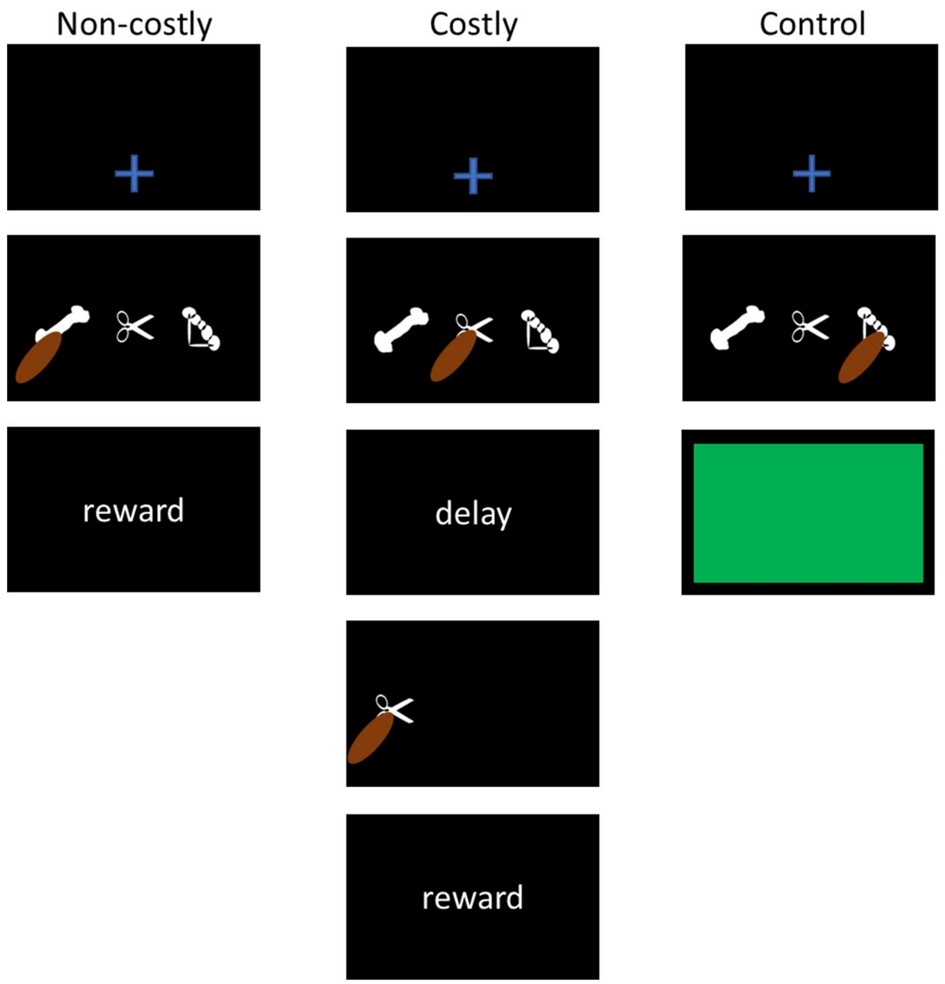

Figure S14: Trials in the preliminary experiment. Non-costly trial: when selected, the stimulus directly delivered the reward. Costly trial: when selected, a delay (between 5000 and 50ms) would be initiated before the same stimulus appeared in a different position. When selected a second time, it triggered the delivery of a reward. Control stimulus: when selected, it resulted in a 3s green screen time out.

Baboons progressed through the experiment when they reached 80% success on one block of 50 trials or after 10 blocks without reaching the criterion. The results (Tab. S8) show that all prosocial monkeys chose the less costly stimuli on all 6 pairs between 4secs and 1 sec.

	5000 ms	4000 ms	3000 ms	2000 ms	1000 ms	500 ms	50 ms
ANGELE	**6**	**6**	**6**	**6**	**6**	5	x
ARIELLE	**6**	**6**	**6**	**6**	**6**	5	**6**
**ATMOSPHERE***	**6**	**6**	**6**	**6**	**6**	x	x
BOBO	**6**	**6**	**6**	x	x	x	x
EWINE	3	2	3	**6**	**6**	3	4
FANA	**6**	**6**	**6**	**6**	5	**6**	**6**
FELIPE	**6**	**6**	**6**	**6**	**6**	**6**	x
**FEYA***	4	**6**	**6**	**6**	**6**	**6**	**6**
HARLEM	**6**	**6**	**6**	**6**	**6**	**6**	**6**
**LIPS***	**6**	**6**	**6**	**6**	**6**	**6**	5
**LOME***	**6**	**6**	**6**	**6**	**6**	**6**	**6**
**MAKO***	**6**	**6**	**6**	**6**	**6**	5	**6**
**MALI***	**6**	**6**	**6**	**6**	**6**	**6**	**6**
**MUSE***	**6**	**6**	**6**	**6**	**6**	**6**	5
NEKKE	5	**6**	**6**	**6**	**6**	**6**	5
PETOULETTE	**6**	**6**	**6**	**6**	**6**	**6**	x
PIPO	x	x	x	x	x	x	x
**VIOLETTE***	5	**6**	**6**	**6**	**6**	**6**	5

Table S8: Results of the preliminary experiment. For each individual and each delay, the number indicates the number of times the non-costly stimuli was chosen above criterion for each of 6 different pairs. Prosocial monkeys are indicated with a star and are in bold font. All 6/6 non-costly stimuli chosen above criterion are indicated in bold, crosses indicate conditions that were not fully completed.

Experiment 3 : Costly-PCT task

The costly-PCT task was similar to Experiment 1, with the exception that the prosocial stimuli had to be touched twice (in different positions) with a delay of one or three seconds (Fig. S17). We first performed a condition with a 1sec delay, then a 3 sec delay, then a 3sec delay ghost condition (similar to the experiment 1 ghost condition but with the costly prosocial choice). We repeated each condition twice with different stimuli and for 10 blocks of 50 trials for each prosocial monkey (except ATMOSPHERE who did not participate reliably in experiments at that time).

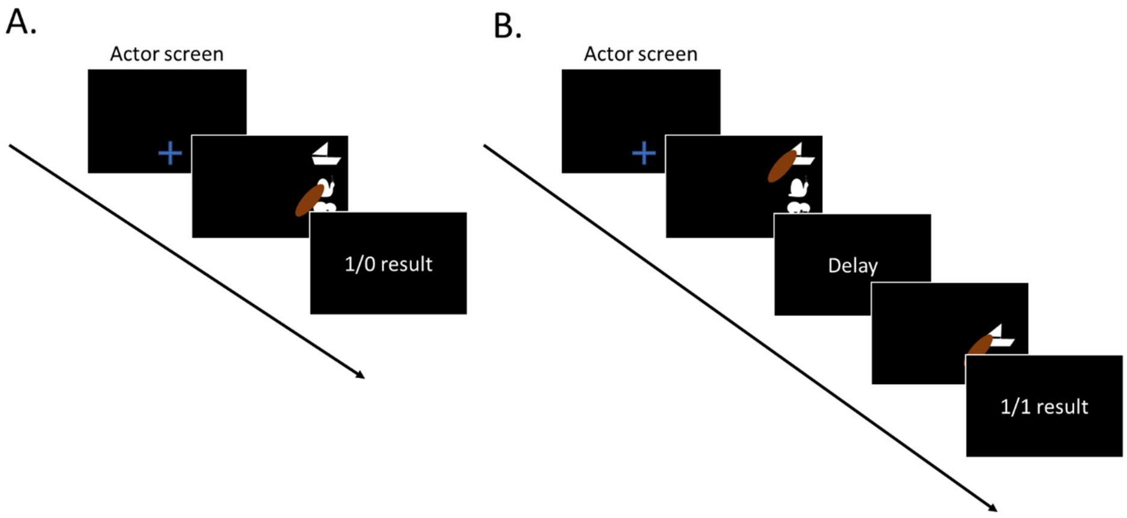

Figure S15: Trials in the costly pct-experiment. A: If the actor chooses the selfish stimulus, a green screen appears immediately on the receiver side, and the actor obtains a reward 1500ms later (as in experiment 1). B: If the actor chooses the prosocial stimulus, the same stimulus appears in a different position after a delay (1000 or 3000ms), and the actor has to touch it again to trigger the reward delivery for the receiver side, followed 1500ms later by a reward for the actor. The outcome of the control stimulus is the same as in experiment 1.

Experiment 3 : Results

We present the results of the 8 previously prosocial individuals, except for ATMOSPHERE, who did not participate reliably in experiments during this period.

In the 1000ms phase, the seven prosocial monkeys reached an average proportion of prosocial choice of 77 % (s.e.: 6 %, min = 48 %, max = 98 %) within the two sets of 10 blocks of trials (Fig. S18), and 74 % (s.e.: 11%, min = 32 %, max = 98 %) in the 3000ms phase (Fig. S18).

During the 1000ms phase, 5/7 monkeys chose the prosocial stimuli above 80% in at least one block of 50 trials for the two sets of stimuli, 2/7 monkeys reached criterion in only one of the two sets. In the 3000ms phase, 4/7 monkeys reached criterion in the two sets, 2/7 in one and 1/7 in none of the two. Compared to experiment 1, this suggests that although prosocial monkeys kept a high rate of prosocial choice, some preferred to choose the non-costly stimulus at some point.

By comparison, in the 3000ms ghost phase, prosocial monkeys reached an average proportion of prosocial choice of 38% (s.e.: 9 %, min = 3%, max = 75%) and 1/7 reached criterion twice, 4/7 reached it once, and 2/7 never reached criterion. These results show that prosocial monkeys were more likely to choose the prosocial option in the 1s and 3s delay condition, compared to the 3s delay ghost control condition (Tab S9). However, the fact that some monkeys persisted in choosing the prosocial stimuli in the ghost condition despite the cost suggest that the delay represented a small cost. Nonetheless, our results demonstrate that monkeys are willing to pay a small cost (a short delay) to sustain cooperation, a cost they are not ready to pay when there is no partner present.

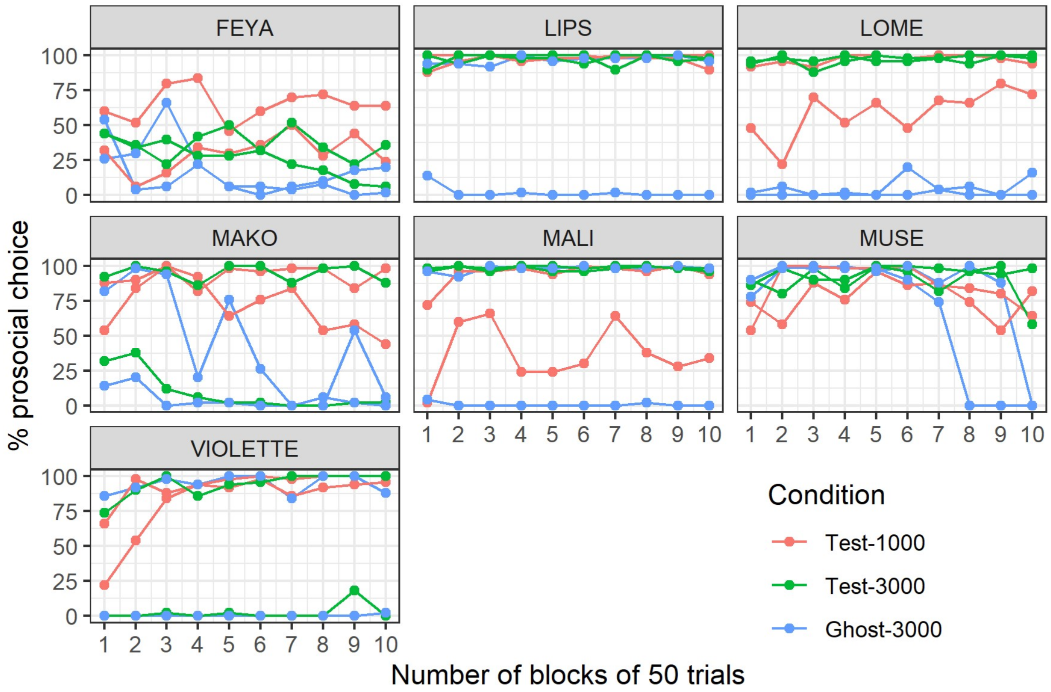

Figure S16: Proportion of prosocial choice in the 1000ms social test phase (red), 3000ms social test phase (green) and ghost control non-social phase (blue). Each line represents a different pair of stimuli.

**Random effect**	**Variance**	**Std.Dev.**		
Intercept (Repetition)	6.16	2.48		
Repetition	4.87	2.21		
Intercept (NbBloc)	2.12	1.46		
NbBloc	0.02	0.13		
**Fixed effect**	**Estimate**	**Std.Error**	**z value**	**Pr(>|z|)**
(Intercept)	-1.02	0.44	-2.35	0.02
Condition : Test - 1000	2.34	0.05	50.42	<0.001
Condition : Test - 3000	2.11	0.05	46.60	<0.001

Table S9: Analysis of prosocial choice depending on delay. GLMM results of the binomial model including as dependent variable the binary choice of stimuli (Non-prosocial = 0, Prosocial = 1), depending on the experimental condition (Test 1000ms delay, Test 3000ms and Ghost 3000ms delay as baseline). We included a random intercept and slope depending on the number of repetitions for the actor and a random intercept and slope depending on the number of blocks for the actor to account for repeated measures (changes in the random structure did not affects the results qualitatively). GLMM analysis was performed using R lme4 package (50).

Effect of dominance

To determine the dominance hierarchy, we followed (*51*), using the data collected in experiment 2 (mean number of supplantation: 216, s.d: 172, min = 9, max= 742). We found no evidence of a relationship between the difference in Elo score between the actor and the receiver and the proportion of prosocial choice made by the actor (Fig. S14). There was no reliable relationship for prosocial monkeys (Fig.S14 A & B) and no group correlation (Fig. S14 C & D; Experiment 1, R-PCT trials, Spearman rho = 0.05, $p<0.49$; Experiment 2, NR-PCT trials, Spearman rho = -0.02, $p=0.88$).

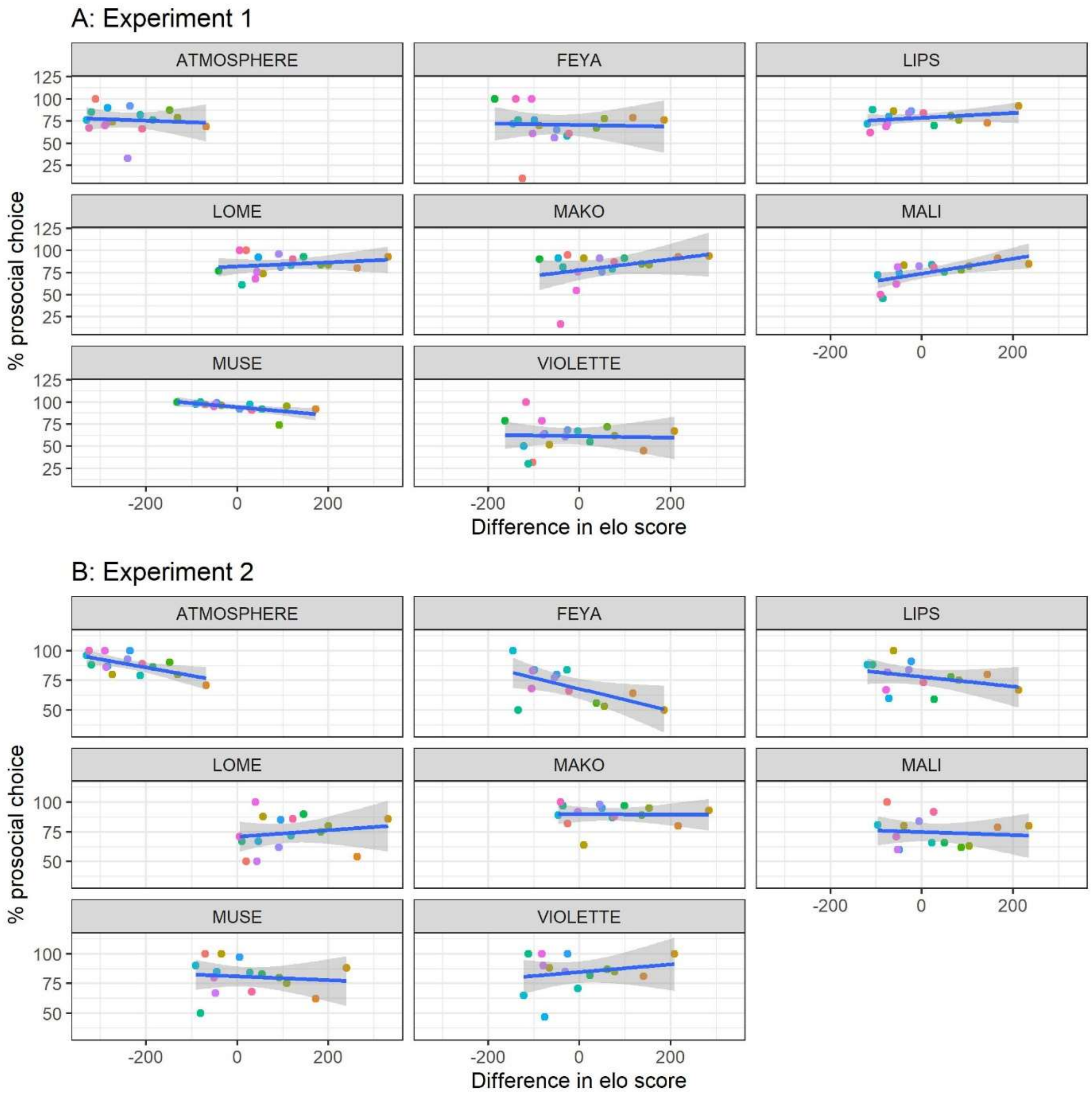
A: Experiment 1
ATMOSPHERE
FEYA
LIPS
LOME
MAKO
MALI
MUSE
VIOLETTE
% prosocial choice
Difference in elo score
B: Experiment 2
ATMOSPHERE
FEYA
LIPS
LOME
MAKO
MALI
MUSE
VIOLETTE
% prosocial choice
Difference in elo score

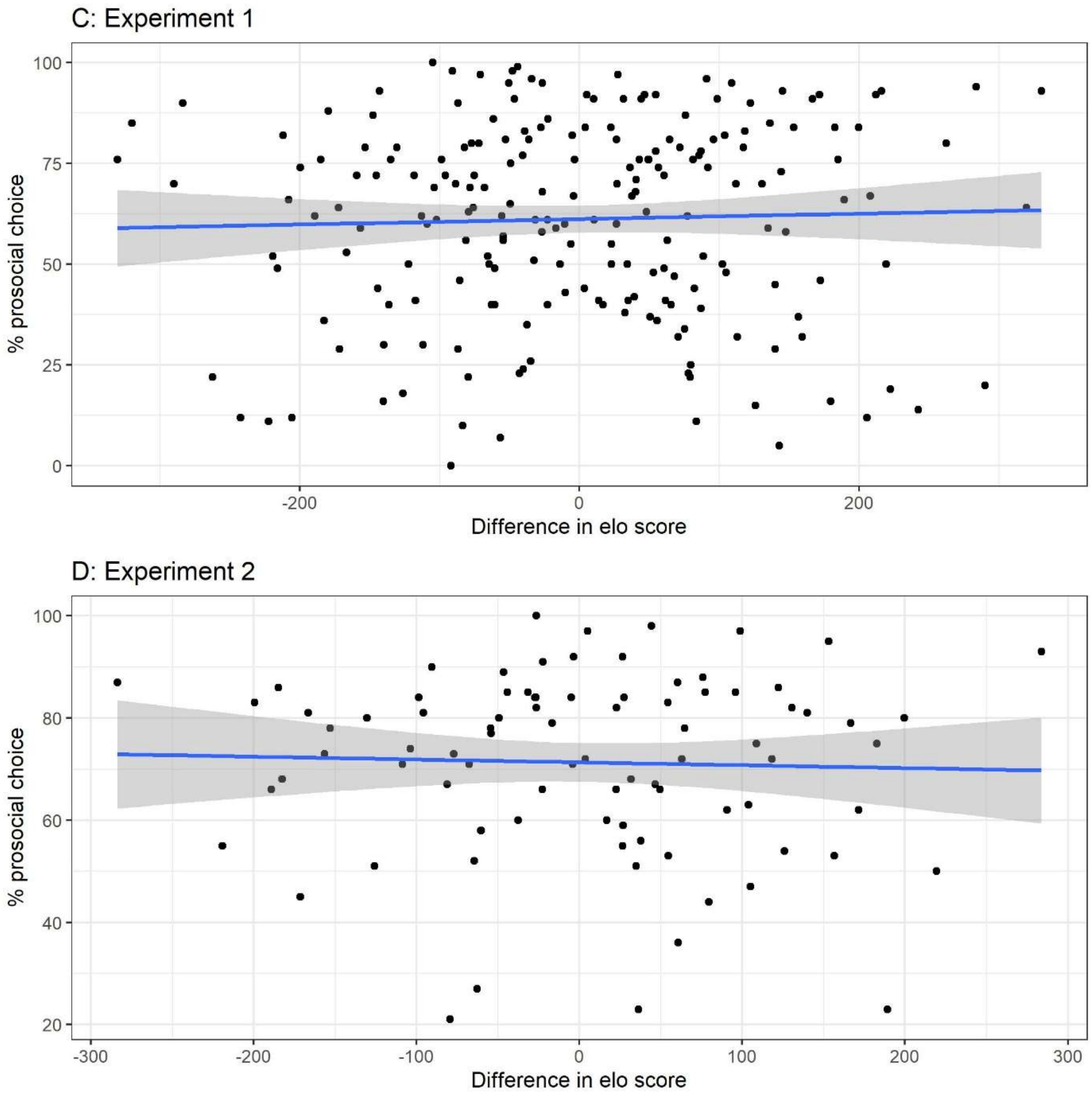

Figure S17: Correlation between the proportion of prosocial choice between pairs of individuals and the dominance rank difference between the actor and the receiver. A: Individual correlation for prosocial monkeys during experiment 1 (R-PCT). B: Individual correlation for prosocial monkeys during experiment 2 (NR-PCT). **C:** Group correlation for every pair of individuals with at least 30 trials during experiment 1 (R-PCT). **D:** Group correlation for every pair with at least 30 trials during experiment 2 (NR-PCT).

<u>Effect of affiliative social network</u>

We conducted behavioral observations throughout Experiment 2 to build the affiliative network of the group following Claidière, Gullstrand, Latouche and Fagot (*52*). We used a five-minute focal sampling method to collect 76.5 hours of behavioral observation including 2560 affiliative behaviors. We found no strong correlation between the affiliative association index (*51*) and the proportion of prosocial choices made by prosocial individuals (Fig S15). There was no reliable relationship for prosocial monkeys (Fig.S15 A & B) and no group correlation (Fig. S15C & D; Experiment 1, R-PCT trials, Spearman rho = 0.06, p=0.50; Experiment 2, NR-PCT trials, Spearman rho = -0.05, p=0.57).

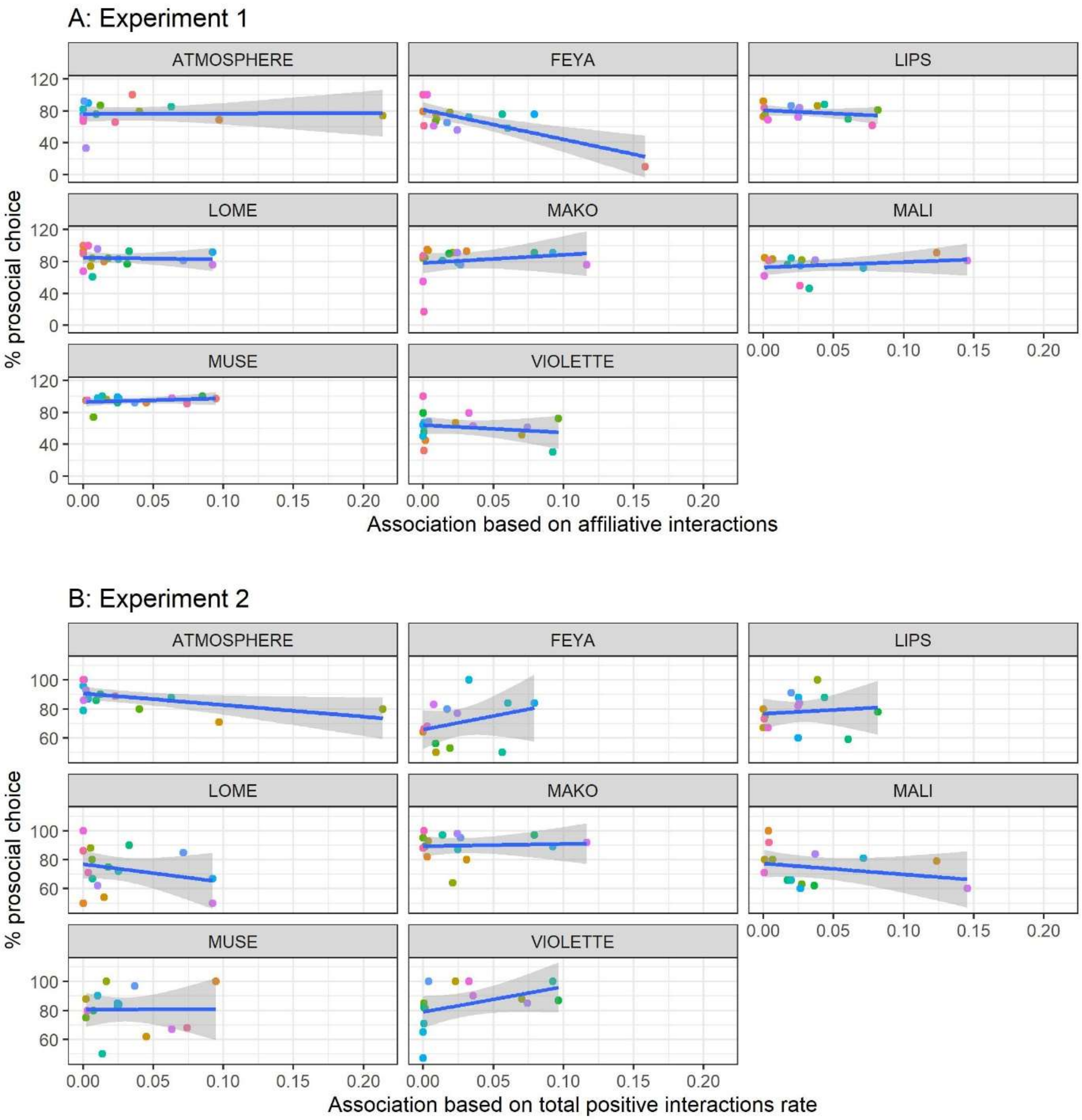
A: Experiment 1
ATMOSPHERE
FEYA
LIPS
LOME
MAKO
MALI
MUSE
VIOLETTE
% prosocial choice
0
40
80
120
0.00
0.05
0.10
0.15
0.20
Association based on affiliative interactions
B: Experiment 2
ATMOSPHERE
FEYA
LIPS
LOME
MAKO
MALI
MUSE
VIOLETTE
% prosocial choice
60
80
100
0.00
0.05
0.10
0.15
0.20
Association based on total positive interactions rate

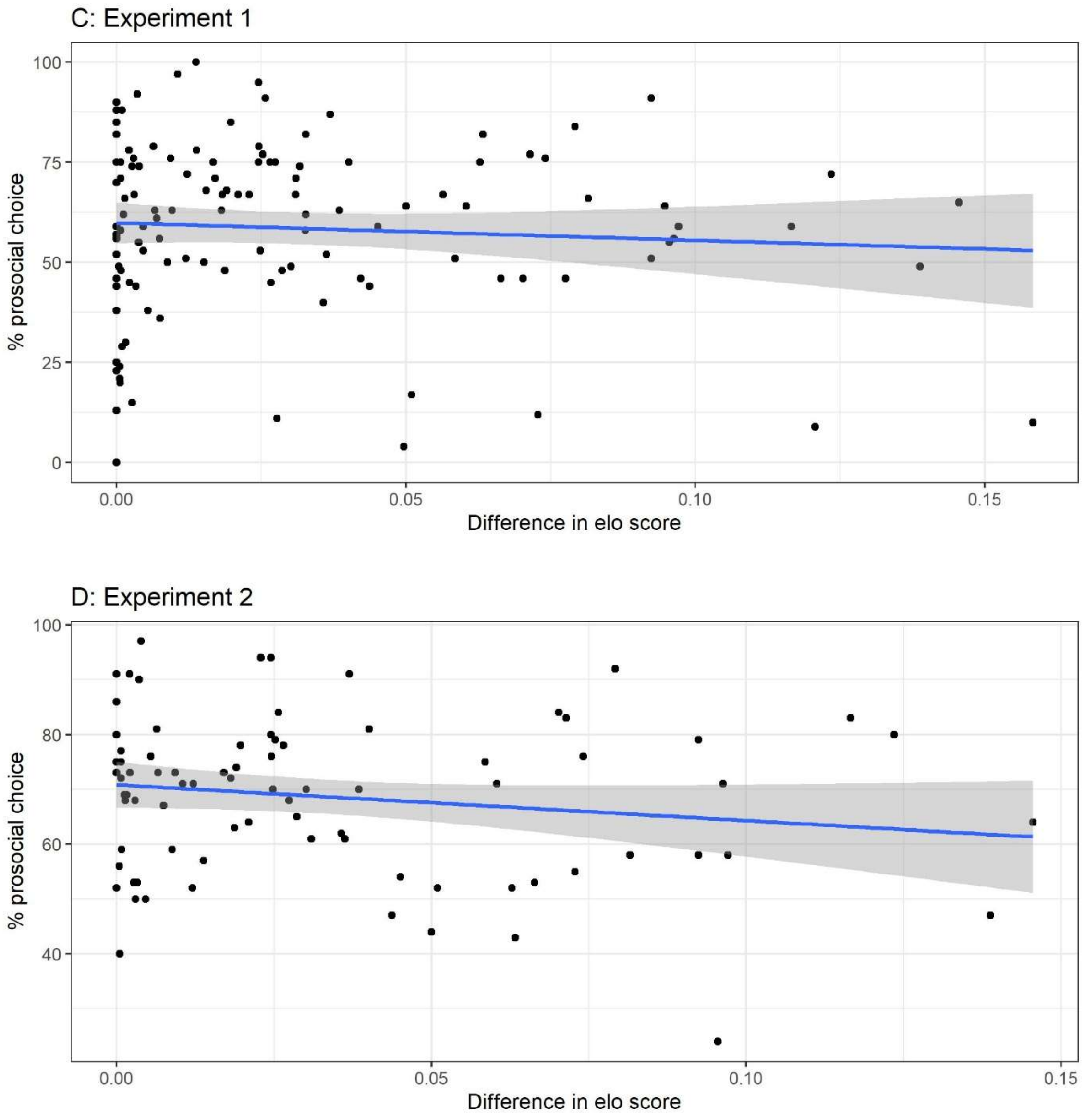

Figure S18: Correlation between the proportion of prosocial choice between pairs of individuals and the association coefficient based on affiliative interactions between the actor and the receiver. A: Individual correlation for prosocial monkeys during experiment 1 (R-PCT). B: Individual correlation for prosocial monkeys during experiment 2 (NR-PCT). **C:** Group correlation for every pair with at least 30 trials during experiment 1 (R-PCT; one extreme point has been removed for the visualization of data but all points were included in the analysis). **D:** Group correlation for every pair with at least 30 trials during experiment 2 (NR-PCT; one extreme point has been removed for the visualization of data but all points were included in the analysis).

Movie S1: Experiment 1 – Ghost condition

Movie S2: Experiment 1 – Test condition

Movie S3: Experiment 2 – Test condition

Movie S4: Experiment 3 – Ghost condition

Movie S5: Experiment 3 – Test condition (3000ms)

REFERENCES AND NOTES

1. S. A. West, C. El Mouden, A. Gardner, Sixteen common misconceptions about the evolution of cooperation in humans. *Evol. Hum. Behav.* **32**, 231–262 (2011).

2. E. Fehr, U. Fischbacher, The nature of human altruism. *Nature* **425**, 785–791 (2003).

3. J. Henrich, R. Boyd, S. Bowles, C. Camerer, E. Fehr, H. Gintis, R. McElreath, M. Alvard, A. Barr, J. Ensminger, N. S. Henrich, K. Hill, F. Gil-White, M. Gurven, F. W. Marlowe, J. Q. Patton, D. Tracer "Economic man" in cross-cultural perspective: Behavioral experiments in 15 small-scale societies. *Behav. Brain Sci.* **28**, 795–815 (2005)

4. S. A. West, A. S. Griffin, A. Gardner, Social semantics: Altruism, cooperation, mutualism, strong reciprocity and group selection. *J. Evol. Biol.* **20**, 415–432 (2007).

5. J. M. Smith, Game theory and the evolution of behaviour. *Proc. R Soc. Lond. B Biol. Sci.* **205**, 475–478 (1979).

6. P. Barclay, Strategies for cooperation in biological markets, especially for humans. *Evol. Hum. Behav.* **34**, 164–175 (2013).

7. S. Debove, J.-B. André, N. Baumard, Partner choice creates fairness in humans. *Proc. Biol. Sci.* **282**, 20150392 (2015).

8. N. Baumard, J.-B. André, D. Sperber, A mutualistic approach to morality: The evolution of fairness by partner choice. *Behav. Brain Sci.* **36**, 59–78 (2013).

9. E. Fehr, B. Rockenbach, Detrimental effects of sanctions on human altruism. *Nature* **422**, 137–140 (2003).

10. R. L. Trivers, The evolution of reciprocal altruism. *Q. Rev. Biol.* **46**, 35–57 (1971).

11. D. G. Rand, M. A. Nowak, Human cooperation. *Trends Cogn. Sci.* **17**, 413–425 (2013).

12. C. L. Apicella, J. B. Silk, The evolution of human cooperation. *Curr. Biol.* **29**, R447–R450 (2019).

13. C. Boesch, H. Boesch, Hunting behavior of wild chimpanzees in the Taï National Park. *Am. J. Phys. Anthropol.* **78**, 547–573 (1989).

14. C. Young, B. Majolo, O. Schülke, J. Ostner, Male social bonds and rank predict supporter selection in cooperative aggression in wild Barbary macaques. *Anim. Behav.* **95**, 23–32 (2014).

15. K. E. Langergraber, D. P. Watts, L. Vigilant, J. C. Mitani, Group augmentation, collective action, and territorial boundary patrols by male chimpanzees. *Proc. Natl. Acad. Sci.* **114**, 7337–7342 (2017).

16. J. B. Silk, S. F. Brosnan, J. Henrich, S. P. Lambeth, S. Shapiro, Chimpanzees share food for many reasons: The role of kinship, reciprocity, social bonds and harassment on food transfers. *Anim. Behav.* **85**, 941–947 (2013).

17. M. Schmelz, J. Call, The psychology of primate cooperation and competition: A call for realigning research agendas. *Philos. Trans. R Soc. Lond. B Biol. Sci.* **371**, 20150067 (2016).

18. J. R. Stevens, F. A. Cushman, M. D. Hauser, Evolving the psychological mechanisms for cooperation. *Annu. Rev. Ecol. Evol. Syst.* **36**, 499–518 (2005).

19. J. R. Stevens, M. D. Hauser, Why be nice? Psychological constraints on the evolution of cooperation. *Trends Cogn. Sci.* **8**, 60–65 (2004).

20. G. Carter, The reciprocity controversy. *Anim. Behav. Cognit.* **1**, 368–386 (2014).

21. A. P. Melis, D. Semmann, How is human cooperation different?. *Philos. Trans. R Soc. Lond. B Biol. Sci.* **365**, 2663–2674 (2010).

22. M. Suchak, T.M. Eppley, M. W. Campbell, R. A. Feldman, L. F. Quarles, F. B. M. de Waal, How chimpanzees cooperate in a competitive world. *Proc. Natl. Acad. Sci. U.S.A.* **113**, 10215–10220 (2016).

23. E. J. C. van Leeuwen, Temporal stability of chimpanzee social culture. *Biol. Lett.* **17**, 20210031 (2021).

24. K. Jensen, The prosocial primate—A critical review, in *Advances in the Study of Behavior*, M. Naguib, J. Brockmann, J. C. Mitani, Eds. (Academic Press, 2016), pp. 387–441

25. K. A. Cronin, Comparative studies of cooperation: Collaboration and prosocial behavior in animals, in *APA Handbook of Comparative Psychology: Basic Concepts, Methods, Neural Substrate, and Behavior*, J. Call, G. M. P. Burghardt, I. M. Snowdon, C. T. Zentall, Eds. (American Psychological Association, 2017), pp. 915–929.

26. K. A. Cronin, Prosocial behaviour in animals: The influence of social relationships, communication and rewards. *Anim. Behav.* **84**, 1085–1093 (2012).

27. R. Noë, Cooperation experiments: coordination through communication versus acting apart together. *Anim. Behav.* **71**, 1–18 (2006).

28. A. Formaux, D. Paleressompoulle, J. Fagot, N. Claidière, The experimental emergence of convention in a non-human primate. *Philos. Trans. R Soc. Lond. B. Biol. Sci.* **377**, 20200310 (2022).

29. J. Fagot, E. Bonté, Automated testing of cognitive performance in monkeys: Use of a battery of computerized test systems by a troop of semi-free-ranging baboons (*Papio papio*). *Behav. Res. Methods* **42**, 507–516 (2010).

30. J. Fagot, D. Paleressompoulle, Automatic testing of cognitive performance in baboons maintained in social groups. *Behav. Res. Methods* **41**, 396–404 (2009).

31. R. S. Mendonça, C. D. Dahl, S. Carvalho, T. Matsuzawa, I. Adachi, Touch-screen-guided task reveals a prosocial choice tendency by chimpanzees (*Pan troglodytes*). *PeerJ* **6**, e5315 (2018).

32. N. Claidière, K. Smith, S. Kirby, J. Fagot, Cultural evolution of systematically structured behaviour in a non-human primate. *Proc. Biol. Sci.* **281**, 20141541 (2014).

33. M. Schmelz, S. Grueneisen, A. Kabalak, J. Jost, M. Tomasello, Chimpanzees return favors at a personal cost. *Proc. Natl. Acad. Sci.* **114**, 7462–7467 (2017).

34. S. Marshall-Pescini, R. Dale, M. Quervel-Chaumette, F. Range, Critical issues in experimental studies of prosociality in non-human species. *Anim. Cogn.* **19**, 679–705 (2016).

35. N. Claidière, A. Whiten, M.C. Mareno, E.J. Messer, S.F. Brosnan, L.M. Hopper, S.P. Lambeth, S.J. Shapiro, N. McGuigan, Selective and contagious prosocial resource donation in capuchin monkeys, chimpanzees and humans. *Sci. Rep.* **6**, 7631 (2015).

36. E. Fehr, H. Bernhard, B. Rockenbach, Egalitarianism in young children. *Nature* **454**, 1079–1083 (2008).

37. A. Formaux, E. O'Sullivan, J. Fagot, N. Claidière, Understanding imitation in *Papio papio*: The role of experience and the presence of a conspecific demonstrator. *Cogn. Sci.* **46**, e13117 (2022).

38. M. Schmelz, S. Grueneisen, M. Tomasello, The psychological mechanisms underlying reciprocal prosociality in chimpanzees (*Pan troglodytes*). *J. Comp. Psychol.* **134**, 149–157 (2020).

39. J. S. Martin, S. E. Koski, T. Bugnyar, A. V. Jaeggi, J. J. M. Massen, Prosociality, social tolerance and partner choice facilitate mutually beneficial cooperation in common marmosets, Callithrix jacchus. *Anim. Behav.***173**, 115–136 (2021).

40. J. J. M. Massen, I. J .A .F. Luyten, B. M. Spruijt, E. H. M. Sterck, Benefiting friends or dominants: prosocial choices mainly depend on rank position in long-tailed macaques (*Macaca fascicularis*). *Primates* **52**, 237–247 (2011).

41. G. Schino, F. Aureli, Reciprocity in group-living animals: Partner control *versus* partner choice. *Biol. Rev. Camb. Philos. Soc.* **92**, 665–672 (2017).

42. R. M. Seyfarth, D. L. Cheney, The origin of meaning in animal signals. *Anim. Behav.* **124**, 339–346 (2017).

43. C. P. van Schaik, J. M. Burkart, Mind the gap: Cooperative breeding and the evolution of our unique features, in *Mind the Gap: Tracing the Origins of Human Universals*, P. M. Kappeler, J. Silk, Eds. (Springer, 2010), pp. 477–496.

44. J. M. Burkart, S. B. Hrdy, C. P. Van Schaik, Cooperative breeding and human cognitive evolution. *Evol. Anthropol.* **18**, 175–186 (2009).

45. S. B. Hrdy, *Mothers and Others: The Evolutionary Origins of Mutual Understanding* (Harvard Univ. Press, 2009), pp. 422.

46. A. D. Colman, K. E. Liebold, J. J. Boren, A method for studying altruism in monkeys. *Psychol. Rec.* **19**, 401–405 (1969).

47. L. M. Hopper, A. Spiteri, S.P. Lambeth, S.J. Schapiro, V. Horner, A. Whiten, Experimental studies of traditions and underlying transmission processes in chimpanzees. *Anim. Behav.* **73**, 1021–1032 (2007).

48. P. M. Fitts, The information capacity of the human motor system in controlling the amplitude of movement. *J. Exp. Psychol.* **47**, 381–391 (1954).

49. I. Blanchette, Y. Marzouki, N. Claidière, J. Gullstrand, J. Fagot, Emotion-cognition interaction in nonhuman primates: Cognitive avoidance of negative stimuli in baboons (*Papio papio*). *Psychol. Sci.* **28**, 3–11 (2017).

50. R. H. Baayen, D. J. Davidson, D. M. Bates, Mixed-effects modeling with crossed random effects for subjects and items. *J. Mem. Lang.* **59**, 390–412 (2008).

51. J. Gullstrand, N. Claidière, J. Fagot, Computerized assessment of dominance hierarchy in baboons (*Papio papio*). *Behav. Res. Methods* **53**, 1923–1934 (2021).

52. N. Claidière, J. Gullstrand, A. Latouche, J. Fagot, Using automated learning devices for monkeys (ALDM) to study social networks. *Behav. Res. Methods* **49**, 24–34 (2017).
